# Impairment of Group I Metabotropic Glutamate Receptors in the Dorsal Striatum of the R451C‐Neuroligin 3 Mouse Model of Autism Spectrum Disorder

**DOI:** 10.1111/jnc.70253

**Published:** 2025-10-13

**Authors:** Maria Meringolo, Martina Montanari, Simona D'Antoni, Giuseppina Martella, Ilham El Atiallah, Giulia Ponterio, Annalisa Tassone, Ingrid Reverte, Daniele Caprioli, Georgios Strimpakos, Luisa Pieroni, Maria Vincenza Catania, Paola Bonsi

**Affiliations:** ^1^ Laboratory of Neurophysiology and Plasticity IRCCS Fondazione Santa Lucia Rome Italy; ^2^ Saint Camillus International University of Health and Medical Sciences Rome Italy; ^3^ Institute for Biomedical Research and Innovation National Research Council Catania Italy; ^4^ Department of Physiology and Pharmacology Sapienza University, and Laboratory of Behavioral Neuropharmacology, IRCCS Fondazione Santa Lucia Rome Italy; ^5^ Institute of Biochemistry and Cell Biology National Research Council Monterotondo Italy; ^6^ Proteomics and Metabolomics Unit IRCCS Fondazione Santa Lucia Rome Italy

**Keywords:** behavior, corticostriatal synapses, electrophysiology, glutamate metabotropic receptors, long‐term synaptic plasticity

## Abstract

Human genetics indicates enrichment of synaptic pathway‐related mutations in Autism Spectrum Disorder (ASD). Accordingly, several preclinical studies have reported synaptic alterations in different brain areas of relevant ASD mouse models. In particular, we previously showed that corticostriatal long‐term synaptic depression is impaired in the dorsal striatum of mice carrying the ASD‐associated R451C mutation in the neuroligin3 (NL3) gene, coding for the postsynaptic protein neuroligin 3. Here, we used behavioral, proteomic, biochemical, and electrophysiological approaches to explore the dorsal striatum‐dependent functions in the R451C‐NL3 knock‐in mouse model of ASD. A detailed behavioral analysis confirmed striatum‐dependent alterations in these mice. We further explored the synaptic function in the dorsal striatum, revealing modifications of the glutamatergic postsynaptic density protein composition and the impairment of different forms of corticostriatal long‐term synaptic plasticity involving the activation of group I metabotropic glutamate receptors, namely activity‐dependent depression and potentiation, and pharmacological 3,5‐DHPG‐induced synaptic depression. Notably, activation of group I metabotropic glutamate receptors was not able to potentiate NMDA receptor‐mediated currents despite unaltered kinetics of the ionotropic receptors. Protein expression levels of type 5 metabotropic glutamate receptors were reduced at striatal synapses. Overall, our findings point to a significant impairment of metabotropic glutamate receptor type 5 signaling in NL3 knock‐in mice, affecting the dorsal striatum circuitry, which is implicated in autism‐related behaviors.

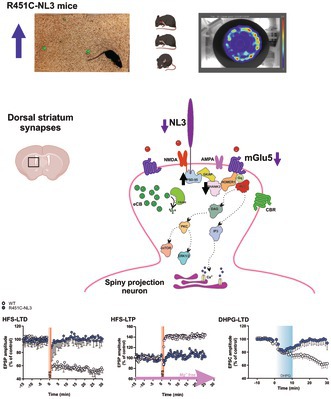

## Graphical abstract

The dorsal and ventral regions of the striatum represent key brain areas for the expression of Autism Spectrum Disorder (ASD) core symptoms. In the present work, we report that different forms of long‐term synaptic plasticity are impaired in the dorsal striatum of a phenotypic mouse model of ASD, carrying the ASD‐associated R451C mutation in the neuroligin 3 gene (R451C‐NL3 mice). We then provide evidence supporting an overall disruption of the glutamatergic postsynaptic density in the dorsal striatum of R451C‐NL3 mice, and a role of group I mGlu receptors impairment in striatal synaptic dysfunction. Abbreviations: DHPG, (S)‐3,5‐dihydroxyphenylglycine; EPSC, excitatory postsynaptic current; EPSP, excitatory postsynaptic potential; HFS, high‐frequency stimulation; LTD, long‐term depression; LTP, long‐term potentiation.

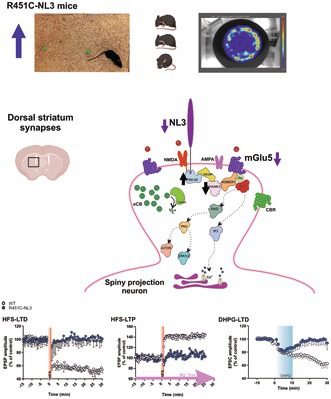

**Abbreviations**
3,5‐DHPG: (S)‐3,5‐dihydroxyphenylglycine
ACEA: arachidonyl‐2′‐chloroethylamide
AM251: 1‐(2,4‐dichlorophenyl)‐5‐(4‐iodophenyl)‐4‐methyl‐N‐1‐piperidinyl‐1H‐pyrazole‐3‐carboxamide
AMPA: α‐amino‐3‐hydroxy‐5‐methyl‐4‐isoxazolepropionic acid
ASD: autism spectrum disorder
CDPPB: 3‐Cyano‐N‐(1,3‐diphenyl‐1H‐pyrazol‐5‐yl)benzamide
CNQX: 6‐Cyano‐7‐nitroquinoxaline‐2,3‐dione
D2R: dopamine type 2 receptor
DARPP‐32: dopamine and cAMP‐regulated neuronal phosphoprotein 2
DEPs: differentially expressed proteins
eCB: endocannabinoid
EPM: Elevated Plus Maze Test
EPSC: excitatory postsynaptic current
EPSP: excitatory postsynaptic potential
ERK: extracellular signal‐regulated kinase
FXS: Fragile X syndrome
GABA: γ‐aminobutyric acid
HFS: high‐frequency stimulation
ID: intellectual disability
IP3: inositol trisphosphate
LTD: long‐term depression
LTP: long‐term potentiation
MAPK: mitogen‐activated protein kinase
MFC: maximum fold change
mGluI: group I metabotropic glutamate
mTOR: mammalian target of rapamycin
NL3: neuroligin3
NMDA: N‐methyl‐D‐aspartic acid
NORT: Novel Object Recognition Test
OCD: obsessive‐compulsive disorder
OF: Open Field Test
PAM: positive allosteric modulator
PI3K: phosphatidylinositol 3‐kinase
PKC: protein kinase C
PMSF: phenylmethanesulfonyl fluoride
PSD: postsynaptic density
PSD‐95: postsynaptic density protein 95
PTX: picrotoxin
PVDF: polyvinylidene fluoride
RDoC: Research Domain Criteria
RRID: Research Resource Identifier
SHANK3: SH3 and multiple ankyrin repeat domains 3
SPN: spiny projection neurons
TTX: tetrodotoxin
WT: wild‐type

## 1 Introduction

Genetic studies indicate a complex and heterogeneous etiology of Autism Spectrum Disorder (ASD). Among commonly affected signaling pathways, those involving synapse formation and regulation of synaptic transmission have been recurrently reported. In particular, over the past 15 years, accumulating evidence has implicated the abnormal expression, signaling, and function of group I metabotropic glutamate (mGluI) receptors in autism (Fatemi and Folsom 2011; Mehta et al. 2011; D'Antoni et al. 2023; Galineau et al. 2023) and autism‐related disorders, such as Fragile X syndrome (FXS) (Ronesi et al. 2012; Rustan et al. 2013; Di Menna et al. 2023), obsessive‐compulsive disorder (OCD) (Ade et al. 2016), and intellectual disability (ID) (D'Antoni et al. 2014; Nisar et al. 2022). mGluI receptors include mGlu1 and mGlu5 subtypes, which are key players in glutamate‐mediated excitatory transmission and overall brain function, as their activity modulates neuronal excitability, intracellular Ca^2+^ signaling, protein synthesis, and dendritic spine maturation (Mango and Ledonne 2023). These metabotropic receptors mediate the action of glutamate either via Gq/11 proteins, phospholipase C, protein kinase C (PKC), diacylglycerol, fostering Ca^2+^ mobilization from internal stores, and finally the MAPK/ERK pathway, or via a Gq/11‐independent pathway involving phosphatidylinositol 3‐kinase (PI3K), Akt, and mammalian target of rapamycin (mTOR) (Potter et al. 2013). Notably, mGlu5 receptors are expressed in the brain from early developmental stages (Catania et al. 2007; Matosin and Newell 2013). The high degree of sequence homology and common signaling mechanisms between mGlu1 and mGlu5 receptors led to the hypothesis that they had redundant functions, but subsequent evidence suggested that these receptors had additional distinct functions (Mannaioni et al. 2001; Pisani et al. 2001; Valenti et al. 2002; Bonsi et al. 2005, 2008), or cross‐talked, acting either cooperatively (Volk et al. 2006) or antagonistically (Poisik et al. 2003; Kramer and Williams 2015). Although mGlu1 and mGlu5 receptors can also be found at presynaptic terminals (Romano et al. 1995), in astrocytes and microglial cells (Spampinato et al. 2018), they are mainly localized in the perisynaptic area of postsynaptic densities (PSDs) (Lujan et al. 1996; Scheefhals et al. 2023), and their activation can lead to cell depolarization and enhanced neuronal excitability. Notably, mGluI receptors play important roles in the expression of long‐lasting forms of synaptic plasticity, long‐term potentiation and depression (LTP and LTD, respectively) (Huber et al. 2000; Huemmeke et al. 2004; Heinke and Sandkühler 2005; Nosyreva and Huber 2006; Anwyl 2009; Gladding et al. 2009; Nicoletti et al. 2011). In particular, LTD expression at corticostriatal synapses depends on the activation of both cortical glutamatergic and nigral dopaminergic inputs to striatal spiny projection neurons (SPNs), leading to both depolarization and activation of mGlu and dopamine type 2 (D2Rs) receptors. This cascade of events leads to the production of endocannabinoids (eCBs), which retrogradely reach and bind presynaptic cannabinoid CB1 receptors, located on glutamatergic terminals, resulting in a long‐term decrease in the probability of neurotransmitter release (Kreitzer and Malenka 2005; Surmeier et al. 2007). Of note, we previously demonstrated the loss of LTD expression at corticostriatal synapses of the dorsal striatum (Martella et al. 2018) in a mouse model carrying the ASD‐associated point mutation R451C in the gene encoding the postsynaptic adhesion protein neuroligin 3 (NL3), the R451C‐NL3 mice. Our study further showed that activation of eCB signaling was able to partially rescue LTD expression at corticostriatal synapses of R451C‐NL3 mice (Martella et al. 2018). This observation led us to hypothesize that the origin of striatal synaptic dysfunction might lie upstream of the eCB production in the LTD induction pathway. In the present study, we therefore examined the expression and function of both mGlu1 and 5 receptor subtypes in the dorsal striatum of R451C‐NL3 mice by using proteomic, biochemical, and electrophysiology approaches. Our findings reveal an impairment of mGlu5 receptor protein expression and function at R451C‐NL3 dorsal striatum synapses and further support the involvement of these receptors in ASD pathophysiology.

## 2 Materials and Methods

### 2.1 Experimental Model

Studies were carried out on adult (P60–P90) R451C‐NL3 knock‐in male mice (B6;129‐Nlgn3tm1Sud/J, RRID:IMSR_JAX:008475) and their wild‐type (WT) littermates. The NL3 gene is located on the X chromosome and ASD cases present a 4:1 prevalence in males versus females; therefore, the study was conducted on male hemizygous mice. All the experiments were approved by the ethics committee of IRCCS Fondazione Santa Lucia and authorized by the Italian Ministry of Health (nr. 492/2019 and 739/2022), in accordance with the Italian D. Lgs 26/2014 and the European Union Directive 2010/63/EU. All efforts were made to minimize the number of animals and their distress and suffering. R451C‐NL3 and WT mice were housed in groups of 4 per cage and maintained with *ad libitum* food and water, with a 12/12 h light/dark cycle and constant temperature (22°C ± 2°C). For genotyping, the R451C mutation was detected after PCR amplification of the insertion site of the first loxP site in the NL3 gene (FW 5′‐TGTACCAGGAATGGGAAGCAG‐3′; RV 5′‐GGTCAGAGCTGTCATTGTCAC‐3′) from genomic DNA extracted from the mouse tail, as previously described (Martella et al. 2018).

### 2.2 Behavioral Assays

Two‐month‐old R451C‐NL3 mice (*N* = 23) and age‐matched WT littermates (*N* = 22) were used for the behavioral analysis. Mice were manipulated once a day for 1 week prior to behavioral testing. After handling habituation, mice were subjected to a battery of behavioral tests as detailed below, each of which was administered once, between 8 a.m. and 4 p.m., on different days. The order of the tests was randomized, except the 3‐chamber test that was always administered at the end, since it requires a period of isolation of the test mouse; hence, it was considered more stressful. To explore the behaviors of the R451C‐NL3 mouse model of ASD, we utilized the Research Domain Criteria (RDoC) framework, which aims at establishing an experimental categorization system where symptoms/domains are classified by anchoring them to the etiological and pathophysiological evidence (https://www.nimh.nih.gov/research/research‐funded‐by‐nimh/rdoc/index.shtml).

#### 2.2.1 Three‐Chamber Social Interaction Test

The 3‐chamber test was used to study sociability and social novelty, as previously described (Farini et al. 2020). The experimental apparatus consisted of a rectangular plexiglass box (60 × 40 × 22 cm) divided into three identical compartments connected by two square openings. One hour prior to the test, animals underwent isolation and acclimatization to the apparatus. The experimental procedure began by placing the test mouse in the central compartment, allowing it to freely explore the three chambers for a duration of 10 min (habituation). Afterwards, for the Sociability phase, an inanimate object was placed in a wire cup in one of the chambers, and an age‐matched stranger WT male mouse in a similar wire cup in the other chamber; the test mouse was allowed to choose to interact with one or the other. After 10 min, the chambers were cleaned, and for the Social Novelty phase (10 min duration), the stranger conspecific was reintroduced in the same chamber, and a novel unfamiliar mouse replaced the object in the first chamber. Each session was recorded using a digital video camera placed above the cage and monitored using Ethovision XT 16 (Noldus). The position of the stranger mouse was randomized between the left and right‐side chambers to rule out possible chamber preferences. The animals used as strangers were habituated to the wire cup for 5 min before the test. Interaction was determined as the moment when the nose of the test mouse was directed toward the wire cup, at less than 2 cm of distance. The Sociability Index evaluates the preference for a conspecific (S) with respect to an object (O), and is measured by the percentage of time spent in close interaction with either S or O on the total time spent interacting with S and with O. The Social novelty index evaluates preference for the novel (N) with respect to the familiar (F) conspecific, measured by the percentage of time spent in close interaction with either N or F on the total time spent interacting with N and with F.

#### 2.2.2 Sucrose Splash Test

The Sucrose Splash Test was performed for investigating anhedonia and motivational behavior, as previously described (Isingrini et al. 2010). Prior to the test, animals were allowed a 1‐h period for acclimatization. Then, they were placed in a standard plexiglass cage (15 × 30 × 20 cm), under red light illumination, and sprayed twice with a 10% sucrose solution onto the dorsal fur. Mice were not deprived of food or water before the test. The delay before initiation and the total duration of the grooming behavior were recorded over a 5‐min period using video recording, with the fully automated video tracking technology Ethovision XT 16 employed for observation and analysis.

#### 2.2.3 Novel Object Recognition Test

The Novel Object Recognition Test (NORT) represents a validated test for assessing recognition memory in rodents that exploit their natural tendency to explore novel items (Cutuli et al. 2020). The experimental arena was a plexiglass 48 × 48 × 24.5 cm square box. The test protocol consisted of three sessions: habituation, training (S1), and test trial (S2), each lasting 5 min. There was a 3 min duration and a 1 h long interval, respectively, between habituation and training, and between S1 and S2 sessions. During the habituation phase, mice were allowed to explore the empty chamber. Subsequently, in the S1 session, they were exposed to two identical objects in the familiar chamber to assess possible preferences for a specific side of the arena (bias). In the final S2 session, one of the S1 familiar objects was replaced by a novel object. The analysis encompasses the measurement of both the total exploration time, calculated as the time spent exploring both the novel (TN) and the familiar (TF) objects during the test phase, and the discrimination index, calculated as DI = (TN—TF)/(TN + TF). The time of direct interaction was evaluated by the time spent sniffing the novel object (TN) or the familiar object (TF). Following each trial, the arena was cleaned with a 10% ethanol solution to mitigate olfactory cues. The movements of the test mice were recorded and tracked using Ethovision XT16.

#### 2.2.4 *Y*‐Maze Test

The *Y*‐maze test was utilized to assess spontaneous alternation behavior and spatial working memory in mice (Kraeuter et al. 2019). The apparatus was in white acrylic plastic, with three identical arms measuring 8 × 30 × 15 cm, set 120° apart. A T‐guillotine door was installed at the gateway of the entrance arm, preventing mouse exit. An entry was counted when a subject placed all four limbs into one of the arms. Each trial lasted 10 min and was recorded and analyzed using Ethovision XT 16. Spatial working memory performance was quantified by calculating the alternation percentage of triads (ABC, BCA, CAB) over the total number of arm entries (Crouch et al. 2018). Additionally, the total number of arm entries was recorded to evaluate exploratory behavior.

#### 2.2.5 Open Field Test

The Open Field Test (OF) was conducted to measure spontaneous locomotor activity in a circular arena (60 cm diameter) during 30 min of observation (Pelosi et al. 2017). This test specifically focused on the normalized distance traveled by mice in the arena central area, defined as a zone 5 cm away from the walls. Data acquisition and analysis were performed using Ethovision XT 16. Following the experimental procedure, a comprehensive analysis was conducted on a range of behavioral parameters. This included locomotor activity (total distance traveled), velocity, time spent in the center, number of entries in the center, and number of defecations.

#### 2.2.6 Self‐Grooming Behavior

During the first 10 min of spontaneous open‐field activity, we additionally examined the self‐grooming behavior, scoring the total number of grooming episodes, defined as complete sequences of cleaning and rubbing the face and snout, licking and stroking flanks and abdomen, anogenital area, and tail, and the total grooming time (Arakawa 2021).

#### 2.2.7 Elevated Plus‐Maze Test

The Elevated Plus Maze (EPM) test was utilized to evaluate anxiety‐like behaviors (Cutuli et al. 2014). The apparatus consisted of a wooden structure comprising two open arms (35 × 6 cm) and two closed arms (35 × 6 × 20 cm) radiating from a central platform. The maze stood 100 cm above the ground. Each mouse was individually placed on the central platform, allowing free exploration for 5 min. Video recordings were acquired using a ceiling‐mounted camera and analyzed with Ethovision XT 16. The number and the latency of entries into open and closed arms (defined by the entry with all four paws) were scored to measure anxiety levels.

#### 2.2.8 Marble Burying Test

The marble burying test was utilized to evaluate repetitive and compulsive behaviors (Angoa‐Pérez et al. 2013). Mice were individually housed in a standard polycarbonate cage measuring 26 × 48 × 20 cm, filled with 5 cm clean bedding. Twenty marbles were tidily laid in four rows on the surface of the bedding. The surface of the bedding was photographed at 5‐min intervals during the 30 min‐long test, to better quantify the number of buried marbles. Marbles were classified as buried if at least three‐quarters were covered by the bedding material.

#### 2.2.9 Accelerating Rotarod Test

The accelerated rotarod test was used to assess coordination and motor learning. The experimental protocol consisted of three training sessions and one test session, delivered along four consecutive days (Rothwell et al. 2014; Sciamanna et al. 2020). The rotarod test was performed using an electronically controlled computerized system (Panlab, Harvard Apparatus), which consists of a rotating cylinder with four lanes (3 cm diameter). Each animal was placed on a rotarod lane. On the first day, mice were acclimated for 30 s to the rod, rotating at 4 rpm constant speed. Then, the rotation velocity was electronically adjusted from 4 to 40 rpm in 300 s with a linear acceleration rate. Each session consisted of three trials with 5‐min intervals. The following parameters were measured: latency to fall (seconds); maximal rotational speed reached before falling (rpm); best performance on the last day of testing (maximal latency to fall) (Sciamanna et al. 2020). Linear regression analyses were used on the dataset obtained from each mouse to derive two key parameters: the intercept as an estimate of initial motor coordination and the slope as a proxy of the learning rate (Rothwell et al. 2014).

### 2.3 Slice Preparation and Electrophysiology Recordings

Forty‐four male R451C‐NL3 mice and 40 wild‐type littermates (P60–P70) were euthanized by cervical dislocation. Corticostriatal slices (300 μm for intracellular or 220 μm for patch‐clamp recordings) were cut with a vibratome in oxygenated artificial cerebrospinal fluid (ACSF; Krebs' solution, in mM: 126 NaCl, 2.5 KCl, 1.3 MgCl_2_, 1.2 NaH_2_PO4, 2.4 CaCl_2_, 10 glucose, 18 NaHCO_3_), as previously described (Martella et al. 2018; El Atiallah et al. 2024). A single slice was transferred into a recording chamber (0.5–1 mL volume), perfused with oxygenated ACSF at constant temperature (32°C–33°C) and flow rate (2–3 mL/min). Conventional intracellular recordings were performed blindly with sharp microelectrodes filled with 2 M KCl (40–60 MΩ), as previously described (Martella et al. 2018; Imbriani et al. 2020). Signals were acquired using an Axoclamp 2B amplifier and pClamp9 software (Molecular Devices). The recorded neurons were identified as spiny projection neurons (SPNs) according to their electrophysiological properties (Martella et al. 2018). Glutamatergic excitatory postsynaptic potentials (EPSPs) were evoked using bipolar electrodes placed in the cortical V–VI layer, in the presence of the GABA_A_ receptor antagonist picrotoxin (PTX, 50 μM) in the perfusion solution. Stimuli were delivered at 0.05 Hz, with 30 μs duration and intensity evoking 70% maximal amplitude of the EPSP. High‐frequency stimulation (HFS), consisting of three trains at suprathreshold intensity (100 Hz frequency, 3 s duration, 20 s interval), was delivered to induce long‐term synaptic plasticity at corticostriatal synapses. Magnesium was omitted from ACSF to remove NMDA receptor blockade and optimize the induction of long‐term synaptic potentiation (LTP) (Calabresi et al. 1992). The EPSP amplitude was continuously recorded for 25 to 40 min after HFS and plotted as a percentage of pre‐HFS mean control EPSP amplitude. Patch‐clamp recordings were performed in the whole‐cell configuration, as previously described (El Atiallah et al. 2024). Striatal SPNs were visualized using BX51WI microscopes (Olympus), with differential interference contrast and standard infrared optics (IR‐DIC), and identified based on their morphology and electrophysiological properties. Electrophysiological signals were detected using Multiclamp 700B amplifiers coupled to pClamp 10.6 software (Molecular Devices). Voltage‐ and current‐clamp recordings were performed with borosilicate glass pipettes (resistance 5–10 MΩ) filled with an internal solution containing (in mM): 125 K^+^‐ gluconate, 10 NaCl, 1 CaCl_2_, 2 MgCl_2_, 0.1 BAPTA, 19 HEPES, 0.3 Na‐GTP, 2 Mg‐ATP; pH 7.35; 300 mOsm. Recordings were discarded if series resistance was outside the 5–30 MΩ range or changed by > 20%. For mGluI receptor‐induced long‐term depression (DHPG‐LTD) experiments, SPNs were clamped at −50 mV holding potential and recorded in the presence of 50 μM PTX (Kreitzer and Malenka 2005). For NMDA current recordings, SPNs were clamped at −70 mV holding potential and recorded in the presence of tetrodotoxin (TTX, 1 μM) plus PTX (Vicidomini et al. 2017). In order to calculate the NMDA/AMPA ratio, recording pipettes were filled with (in mM): 120 CsMeSO_3_, 15 CsCl, 8 NaCl, 10 TEA‐Cl, 2–5 QX‐314, 10 HEPES, 0.2 EGTA, 2 Mg‐ATP, and 0.3 NaGTP; pH adjusted to 7.3 with CsOH; 300 mOsm (Sciamanna et al. 2012). Excitatory postsynaptic currents (EPSCs) were initially recorded at −70 mV holding potential, in the presence of PTX. Then, the holding potential was brought to +40 mV, and NMDA‐mediated EPSCs were measured in the presence of PTX plus the AMPA receptor antagonist CNQX (10 μM). Drugs were bath applied by switching the perfusion solution to the drug‐containing solution using a three‐way tap syringe. PTX (cat. no. 1675) was from Sigma‐Aldrich (Italy). (S)‐3,5‐Dihydroxyphenylglycine (3,5‐DHPG, cat. no. 0805), 3‐Cyano‐N‐(1,3‐diphenyl‐1H‐pyrazol‐5‐yl)benzamide (CDPPB, cat. no. 3235), arachidonyl‐2′‐chloroethylamide (ACEA, cat. no. 1319), 1‐(2,4‐dichlorophenyl)‐5‐(4‐iodophenyl)‐4‐methyl‐N‐1‐piperidinyl‐1H‐pyrazole‐3‐carboxamide (AM251, cat. no. 1117), N‐methyl‐D‐aspartic acid (NMDA, cat. no. 0114), 6‐Cyano‐7‐nitroquinoxaline‐2,3‐dione disodium (CNQX disodium salt, cat. no. 1045), and TTX (cat. no. 1069) were purchased from Tocris‐Cookson (UK).

### 2.4 Western Blot

Western blot of striatal lysates was performed as previously described (Bonsi et al. 2019; El Atiallah et al. 2024). Dorsal striata were collected from 10 R451C‐NL3 mice and respective 8 WT littermates, euthanized by cervical dislocation. Samples were homogenized in cold extraction buffer: 50 mM Tris–HCl pH 7.4, 150 mM NaCl, 1% Triton X‐100, 0.25% Na deoxycholate, 5 mM MgCl_2_, 0.1% SDS, 1 mM EDTA, and 1% protease inhibitor cocktail (Sigma‐Aldrich). Afterwards, the samples were sonicated, kept on ice for 1 h, and centrifuged (13 000 rpm, 15 min, 4°C). The protein content was quantified with the Bradford assay (Bio‐Rad). DTT and NuPage LDS sample buffer (cat. no. D1532 and NP0007, Invitrogen, Life Technologies) were added to protein extracts, and striatal lysates were denatured (95°C, 5 min) and loaded onto 8%–10% SDS‐PAGE gels. Gels were blotted onto a polyvinylidene fluoride (PVDF) membrane, which was then incubated overnight at 4°C with the following primary antibodies: rabbit anti‐mGlu1 receptor (1:1000, cat. no. D5H10–12 551, Cell Signaling), rabbit anti‐mGlu5 receptor (1:5000, cat. no. ab76316, Abcam), rabbit anti‐NMDA receptor subunit 2A (GluN2A) (1:1000, cat. no. 4205, Cell Signaling), rabbit anti‐NMDA receptor subunit 2B (GluN2B) (1:1000, cat. no. 4207, Cell Signaling), rabbit anti‐AMPA receptor subunit 1 (GluA1) (1:1000, cat. no. PA5‐32425, Invitrogen), rabbit anti‐AMPA receptor subunit 2 (GluA2) (1:1000, cat. no. AB1768‐I, Sigma Aldrich), rabbit anti‐SHANK3 (1:1000, cat. no. GTX133163, GeneTex), mouse anti‐PSD95 (1:20000, cat. no. MAB1598, Millipore), and rabbit anti‐NL3 (1:8000, cat. no. GTX131085, GeneTex). Mouse anti‐β actin (1:10.000, cat. no. A5441, Sigma‐Aldrich) was incubated for 1 h at room temperature (RT). Anti‐mouse and anti‐rabbit horseradish peroxidase (HRP)‐conjugated secondary antibodies were used (GE Healthcare). EveryBlot Blocking Buffer (cat. no. 12010020, Biorad) and Tris–HCl washing buffer (cat. no. 15567027, Life Technologies) were used. Immunodetection was performed with ECL Prime Cytiva reagent (cat. no. RPN2232, Sigma‐Aldrich) and iBright CL1000 instrument (Thermo Fisher). Quantification was achieved by ImageJ software (NIH).

### 2.5 Preparation and Western Blot Analysis of Synaptic Plasma Membranes

Synaptosomes were obtained from the dorsal striata of 8 three‐month‐old control (WT) and 10 R451C‐NL3 mice, sacrificed by cervical dislocation. Frozen brain samples were allowed to thaw on ice, weighed, and homogenized in 10% (w/v) 0.32 M sucrose buffer containing ethylenediaminetetraacetic acid (EDTA 1 mM, Sigma), Tris–HCl (10 mM, pH 7.4, Sigma), phenylmethanesulfonyl fluoride (PMSF 0.5 mM, Sigma), and protease inhibitor cocktail (Roche). The homogenate was centrifuged for 10 min at 1000 × *g* (4°C) to separate the nuclear pellet. The supernatant was then centrifuged for 30 min at 18000 × *g* (4°C), and the resulting pellet was resuspended in 0.32 M sucrose buffer, layered on top of a discontinuous sucrose gradient (0.8, 1.0, 1.2 M), and centrifuged for 2 h at 75000 × *g* (4°C). The synaptic plasma membrane fractions were stratified at the interface between 1/1.2 M sucrose. The recovered synaptosomes were diluted 1:2 in cold sucrose buffer and centrifuged for 1 h at 40000 × *g* (4°C). The pellets were resuspended in Hepes buffer (0.5 mM PMSF, 10 mM Hepes, and protease inhibitor cocktail; pH 7.4). A 5 μL volume of the suspension was used to determine the protein content by using the BCA method, and the remaining was stored at −80°C until use. For Western Blot analysis, 10 μg of proteins from WT and R451C‐NL3 samples were denatured in sample buffer (2X) at 37°C for 5 min, separated by 7.5% SDS‐polyacrylamide gels, and transferred onto nitrocellulose membranes (Hybond C‐extra, Amersham Biosciences) with a trans‐blot semi‐dry transfer cell (Bio‐Rad). The filters were processed with the Western Breeze Chemiluminescent Immunodetection System kit (Invitrogen), as indicated by the manufacturer. In brief, filters were blocked for 30 min with blocking solution and incubated overnight with the following primary antibodies: anti‐mGlu5 receptor (rabbit 1:2000, cat. no. ab76316, Abcam), anti‐mGlu1α (mouse, 1:1000, cat. no. G209‐488 BD Pharmingen), anti‐NL3 (rabbit 1:1000, cat. no. SYSY 129113, Synaptic Systems), and anti‐β‐actin (mouse 1:1000, cat. no. 3700, Cell Signaling). Alkaline phosphatase‐conjugated secondary anti‐rabbit or anti‐mouse antibodies from the Invitrogen kit were used. Chemiluminescence was detected and quantified by the VersaDoc 4000 Imaging System (Bio‐Rad). After detection, membranes incubated with anti‐β‐actin antibody were washed and incubated with an anti‐Homer pan antibody (rabbit 1:1000, cat. no. Sc‐15 321, Santa Cruz). After washing, membranes were incubated with a specified secondary antibody and then the signal was detected and quantified, as previously described.

### 2.6 Statistical Analysis

The number of experimental subjects for electrophysiological and biochemical experiments was determined based on our previous studies (Martella et al. 2018; Sciamanna et al. 2020; Bonsi et al. 2019; El Atiallah et al. 2024). For behavioral experiments, sample size was calculated with GPower 3.1 (*t* tests; difference between two independent means; a priori power analysis—compute required sample size; effect size *d* = 0.875; *α* error probability = 0.05; power = 0.8) based on published data (Rothwell et al. 2014). Behavioral and biochemical experiments were analyzed blind to genotype; electrophysiology experiments were performed and analyzed blind to experimental variables. Analysis of electrophysiological experiments was performed offline using Clampfit 10 software (pClamp 10, Molecular Devices). Statistical analysis was performed with Prism software (GraphPad). The normality of data distribution was investigated with the Shapiro–Wilk test. The ROUT method was utilized for the identification of possible outliers, and no data were excluded. For single comparisons of parametric data, paired or unpaired *t*‐test was used, whereas non‐parametric data were analyzed by Mann–Whitney tests. One‐way ANOVA followed by Sidak's multiple comparison test was utilized to analyze multiple datasets with normal distribution. Two‐way ANOVA, followed by Sidak's multiple comparison test, was used for time‐course analysis of data from open field and rotarod tests and interaction time comparisons in the 3‐chamber test. Multiple t test followed by FDR multiple comparison correction was used to compare the data obtained from the marble test. Statistical significance was set at *p* < 0.05 (*p < 0.05, **p < 0.01). Data are presented as mean ± standard error of the mean (SEM). For electrophysiology, each cell was recorded from a different brain slice. The number of biological replicates is reported with N for animals and n for cells.

## 3 Results

### 3.1 The R451C Mutation in the Neuroligin 3 Gene Induces Dorsal Striatum‐Dependent ASD‐Like Behaviors in Mice

In the present study, we conducted a comprehensive behavioral analysis of the stereotypic and motor behaviors dependent on the dorsal striatum in R451C‐NL3 mice.

**FIGURE 1:**
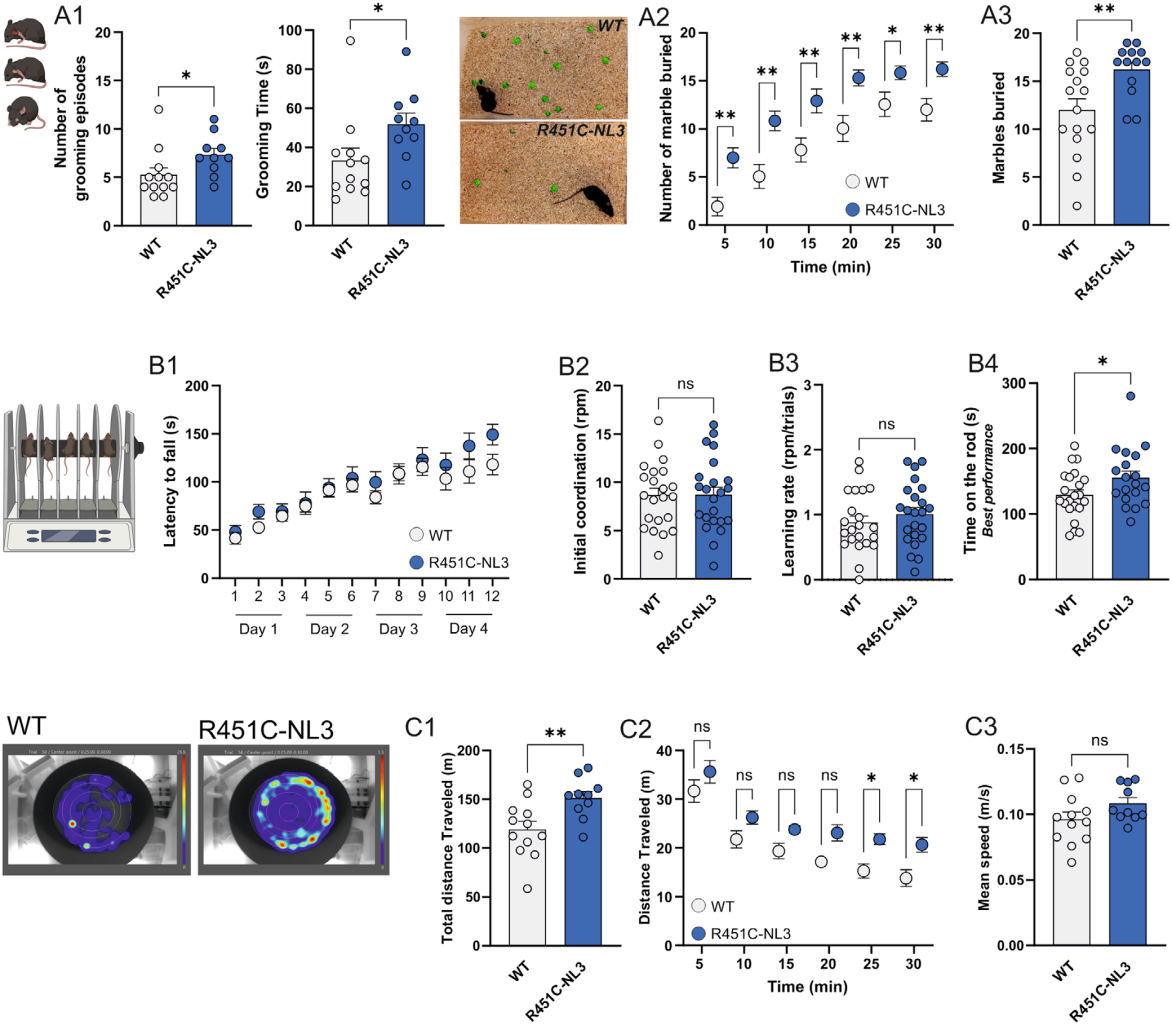
Behavioral characterization of R451C‐NL3 mice. *Repetitive behaviors*: (A1) Summary plot of the total number and duration of self‐grooming events. Only the complete sequences of body grooming were considered, as illustrated by the cartoon (*inset*). (A2) Time course of the average number of marbles buried during a 30 min‐long session, subdivided in 5 min‐long time bins. WT mice: 5 min: 1.94 ± 0.97; 10 min: 5.06 ± 1.24; 15 min: 7.81 ± 1.25; 20 min: 10.06 ± 1.37; 25 min: 12.56 ± 1.27; 30 min: 12.00 ± 1.17; R451C‐NL3 mice: 5 min: 7.00 ± 1.04; 10 min: 10.85 ± 1.02; 15 min: 12.92 ± 1.24; 20 min: 15.31 ± 0.83; 25 min: 15.85 ± 0.71; 30 min: 16.23 ± 0.75. (A3) Bar chart reporting the total number of marbles buried during the entire session by WT (*N* = 16) and R451C‐NL3 (*N* = 13) mice. *Motor function*: (B1) Motor coordination, measured as the time to fall from the accelerating rotarod (4–40 rpm) during 3 daily sessions, along 4 consecutive days. The final speed of rotation reached was used to calculate the initial coordination (B2) and the learning rate (B3). (B4) Bar chart reporting the best performance obtained on the last day. (C) *Left*. Representative heat maps of a WT and a R451C‐NL3 mouse, created by Ethovision software at the end of the open field session. (C1) Average total distance (meters) traveled in the arena by WT (*N* = 12) and R451C‐NL3 (*N* = 10) mice. (C2) Average traveled distance (meters) measured in 5‐min long time bins during a 30‐min session. (C3) Average speed (m/s) of WT and R451C‐NL3 mice during the 30‐min session. Mean ± SEM of data is reported. In the bar graphs, each dot represents a single measurement. (ns) *p* > 0.05; *p ≤ 0.05; **p ≤ 0.01.

Firstly, we assessed repetitive behaviors of R451C‐NL3 mice by evaluating self‐grooming during spontaneous activity in the open field test. Normal grooming behavior involves brief bouts lasting seconds to minutes of scratching and fur cleaning using the forelimbs (Kim et al. 2016). We observed a significant increase in the number and duration of complete self‐grooming sequences in R451C‐NL3 mice, with respect to WT littermates, indicating the expression of a stereotypic behavior in mutants (Figure 1A1; *left*, WT = 5.25 ± 0.729, *N* = 12; R451C‐NL3 = 7.30 ± 0.668, *N* = 10; Mann–Whitney test, *U* = 26, *p = 0.022; *right*, WT = 33.26 ± 6.33 s, *N* = 12; R451C‐NL3 = 51.79 ± 5.77 s, *N* = 10; Mann–Whitney test, *U* = 22, *p = 0.011). We then utilized, for the first time in the R451C‐NL3 mouse model, the marble‐burying, a consolidated test in the study of rodent stereotypic behaviors (Mehta et al. 2011; Amodeo et al. 2012; Angoa‐Pérez et al. 2013). Interestingly, this test highlighted a significant increase in the digging and burying behavior of mutants, compared to WT mice (Figure 1A2,A3), further supporting an increase in repetitive behaviors. Specifically, Multiple *t* test analysis followed by FDR multiple comparison correction showed a significant increase in the number of marbles buried by R451C‐NL3 with respect to control mice at each time interval evaluated during the observation period (Figure 1A2; 0–5 min, **p = 0.0015; 5–10 min, **p = 0.0017; 10–15 min, **p = 0.0080; 15–20 min, **p = 0.0046; 20–25 min, *p = 0.0436; 25–30 min, **p = 0.0077; df = 27). Overall, throughout the test duration, R451C‐NL3 mice buried significantly more marbles compared to their WT littermates (Figure 1A3; WT = 12.0 ± 1.17, *N* = 16; R451C‐NL3 = 16.23 ± 0.75, *N* = 13; Mann–Whitney test, *U* = 40, **p = 0.004).

Motor behaviors, which are also based on the dorsal striatum, were then investigated. First, we examined motor coordination and learning with the accelerated rotarod test, a task that requires formation and consolidation of a repetitive motor routine (Sciamanna et al. 2012; Rothwell et al. 2014). The performance improved similarly across the training sessions in mutant and control mice (Figure 1B1; two‐way ANOVA, *F*(4.707, 127.1)_time_ = 37.08, ****p < 0.0001). Neither the genotype (two‐way ANOVA *F*(1, 27)_genotype_ = 1.166, *p* = 0.289), nor the interaction between genotype and training (two‐way ANOVA *F*(11, 297)_interaction_ = 1.190, *p* = 0.293) had a significant impact on the observed improvement. The linear regression analysis did not detect any significant differences between the genotypes in the initial coordination (Figure 1B2; WT = 8.67 ± 0.72 r.p.m., *N* = 22; R451C‐NL3 = 8.71 ± 0.78 r.p.m., *N* = 23; unpaired *t*‐test, *t*(43) = 0.041, *p* = 0.967) or learning rate (Figure 1B3; WT = 0.88 ± 0.099, *N* = 22; R451C‐NL3 = 1.01 ± 0.099, *N* = 23; unpaired *t*‐test, *t*(43) = 0.915, *p* = 0.365). However, R451C‐NL3 mice showed a significantly better performance on the last day (day 4) with respect to WT littermates (Figure 1B4; WT = 129.00 ± 7.90 s, *N* = 22; R451C‐NL3 = 155.40 ± 10.29 s, *N* = 19; unpaired *t*‐test, *t*(39) = 2.065, *p = 0.046), in accordance with a previous report (Rothwell et al. 2014), suggesting a more efficient consolidation of a repetitive motor routine. To determine the overall motor activity level, we performed the open field test. R451C‐NL3 mice showed a significant increase in the total horizontal distance traveled (Figure 1C1; WT = 119.00 ± 8.38 m, *N* = 12; R451C‐NL3 = 151.20 ± 6.64 m, *N* = 10; unpaired *t*‐test, *t*(20) = 2.920, **p = 0.009), and in the horizontal distance traveled in the last two time‐intervals of the session (Figure 1C2); Sidak's multiple comparisons test 20–25 min, *p = 0.011; 25–30 min, *p = 0.043; Two‐way ANOVA: main effect of time (*F* (2.116, 42.31) = 57.53, ****p < 0.0001), and genotype (*F* (1,20) = 8.528, **p = 0.0085), indicating hyperactive behavior. However, the average speed detected during the open field test was not significantly different between the two genotypes (Figure 1C3; WT = 0.097 ± 0.005 m/s, *N* = 12; R451C‐NL3 = 0.108 ± 0.004 m/s, *N* = 10; unpaired *t*‐test, *t*(20) = 1.664, *p* = 0.112), suggesting that the increased locomotor activity of R451C‐NL3 mice might be caused by striatal circuitry impairments affecting the ability to set goals, maintain a direction, and follow a smooth trajectory (Vernazza‐Martin et al. 2005).

In order to further validate R451C‐NL3 mice as a reliable ASD model in our setting, we also evaluated social behaviors with the three‐chamber test. During the sociability session, the mutant mice showed no preference for the social over the non‐social stimulus (Figure S1A1), also evidenced by a reduced social preference index (Figure S1A2). Similarly, during the following social novelty session, R451C‐NL3 mice displayed no difference in the interaction time spent with either the familiar or the novel mouse (Figure S1A3). Hence, R451C‐NL3 mice showed a reduced novel preference index compared to control animals (Figure S1A4). Additionally, we found that mice carrying the R451C‐NL3 mutation showed a reduced self‐grooming activity compared to WT littermates in the sucrose splash test (Figure S1B1,B2), indicating a reduced motivation. We therefore did not find significant alterations in cognitive function, investigated with the novel object recognition and the *Y*‐maze test (Figure S1C1,C2,D1,D2). Similarly, we did not observe changes in anxiety‐related behaviors in R451C‐NL3 mice, measured in the open‐field and the elevated‐plus maze test (Figure S1E1–E3,F1,F2).

### 3.2 The R451C Mutation Impairs the Expression of Corticostriatal Bidirectional Synaptic Plasticity

**FIGURE 2:**
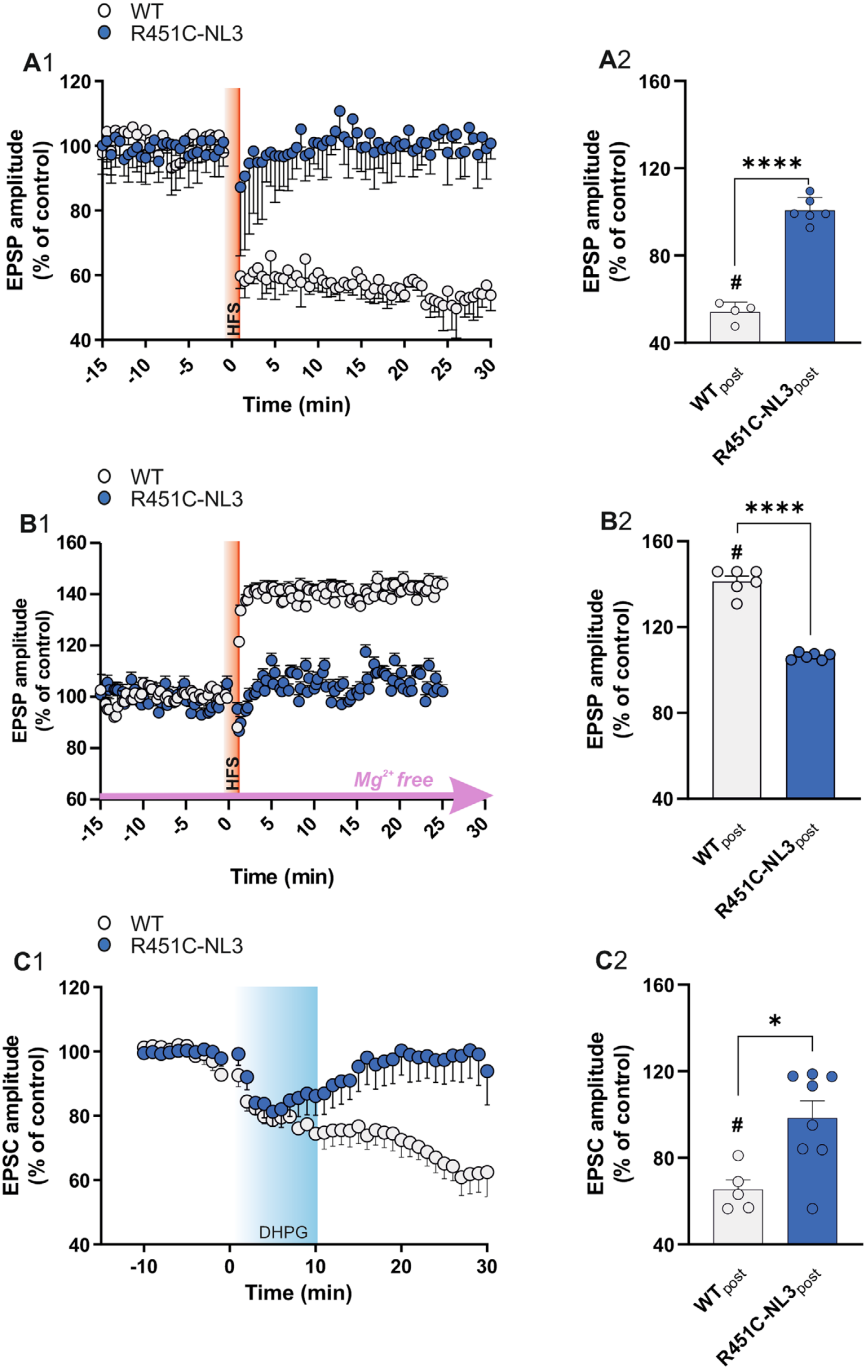
Analysis of bidirectional corticostriatal long‐term synaptic plasticity. (A1) Graph reporting the time course of changes induced by HFS in the amplitude of EPSP evoked in SPNs by cortical stimulation. Data are plotted as mean ± SEM. (A2) The bar graph shows average EPSP amplitudes measured after HFS (POST, last 5 min of recording), reported as a percentage of the mean amplitude at baseline (5 min before HFS). (B1) Summary plot reporting the time course of corticostriatal LTP induced by HFS stimulation in the absence of magnesium in the perfusion solution during intracellular recordings from SPNs. Data are represented as mean ± SEM. (B2) The bar graph shows average EPSP amplitudes measured after HFS (POST, last 5 min of recording), reported as a percentage of the mean amplitude at baseline (5 min before HFS). (C1) Summary plot showing the time course of pharmacological LTD induced in patch‐clamp recordings by brief bath application of DHPG (50 μM, 10 min). Data are represented as mean ± SEM. (C2) The bar graph reports average EPSC amplitudes measured after DHPG (POST, last 5 min of recording), reported as a percentage of the mean amplitude at baseline (5 min before DHPG). Each dot represents a single measurement. Means ± SEM of data are reported. Paired *t*‐test comparison of PRE‐ versus POST‐HFS values: #*p* ≤ 0.05. Unpaired *t*‐test comparison of WT versus R451C‐NL3 POST‐HFS values: *p ≤ 0.05; ****p ≤ 0.0001.

Corticostriatal synapses express different forms of long‐term bidirectional plasticity, namely potentiation (LTP) and depression (LTD), that can be induced experimentally by either high‐frequency stimulation (HFS) of afferent fibers (Calabresi et al. 1992), or by agonist‐induced mGluI receptors activation (Kreitzer and Malenka 2005). As we previously showed (Martella et al. 2018), delivery of HFS to cortical fibers was not able to induce an activity‐dependent LTD at R451C‐NL3 dorsal striatum synapses (Figure 2A1,A2; *EPSP amplitude post‐HFS*: WT_POST_ = 54.10 ± 2.28% of pre‐HFS control, *n* = 4; paired *t*‐test, *t*(3) = 20.610, ***p = 0.0002; R451C‐NL3_POST_ = 100.70% ± 2.38% of pre‐HFS control, *n* = 6; paired *t*‐test, *t*(5) = 0.552, *p* = 0.6045; WT_POST_ versus R451C‐NL3_POST_ unpaired *t*‐test, *t*(8) = 13.380, ****p < 0.0001). We therefore investigated other forms of long‐term synaptic plasticity that can be induced at corticostriatal synapses. We found that activity‐dependent long‐term potentiation (HFS‐LTP) is also impaired in R451C‐NL3 dorsal striatum. In fact, delivery of the induction protocol in R451C‐NL3 slices failed to induce an LTP of similar amplitude to the potentiation observed in WT (Figure 2B1,B2; *EPSP amplitude post‐HFS*: WT_POST_ = 141.40% ± 2.43% of pre‐HFS control, *n* = 6; paired *t*‐test, *t*(5) = 17.040, ****p < 0.0001; R451C‐NL3_POST_ = 106.40% ± 0.63% of pre‐HFS control, *n* = 6; paired *t*‐test, *t*(5) = 10.040, ***p = 0.0002; WT_POST_ vs. R451C‐NL3_POST_ unpaired *t*‐test, *t*(10) = 10.930, ****p < 0.0001). We then examined the expression of mGluI‐dependent pharmacological LTD in the R451C‐NL3 striatum. Bath application of 50 μM (RS)‐3,5‐dihydroxyphenylglycine (DHPG; 10 min) resulted in a sustained LTD of the excitatory postsynaptic currents (EPSCs) in WT slices. Conversely, the same protocol in R451C‐NL3 slices induced only a transient decrease of EPSC amplitude, which then returned to levels not significantly different from baseline (Figure 2C1,C2; *EPSC amplitude post‐DHPG*: WT_POST‐DHPG_ = 65.35 ± 4.53% of pre‐DHPG control, *n* = 5, paired *t*‐test, *t*(4) = 7.652, **p = 0.0016; R451C‐NL3_POST‐DHPG_ = 98.31% ± 0.53% of pre‐DHPG control, *n* = 8, paired *t*‐test, *t*(7) = 0.206, *p* = 0.843; WT_POST‐DHPG_ vs. R451C‐NL3_POST‐DHPG_, unpaired *t*‐test, *t*(11) = 3.050, *p = 0.011).

These data demonstrate an overall impairment of different forms of long‐term synaptic plasticity, encompassing activity‐dependent and pharmacologically induced, in the dorsal striatum of R451C‐NL3 mice.

### 3.3 Exogenous Activation of CB1 Receptors Rescues the Expression of mGluI‐Induced LTD.

**FIGURE 3:**
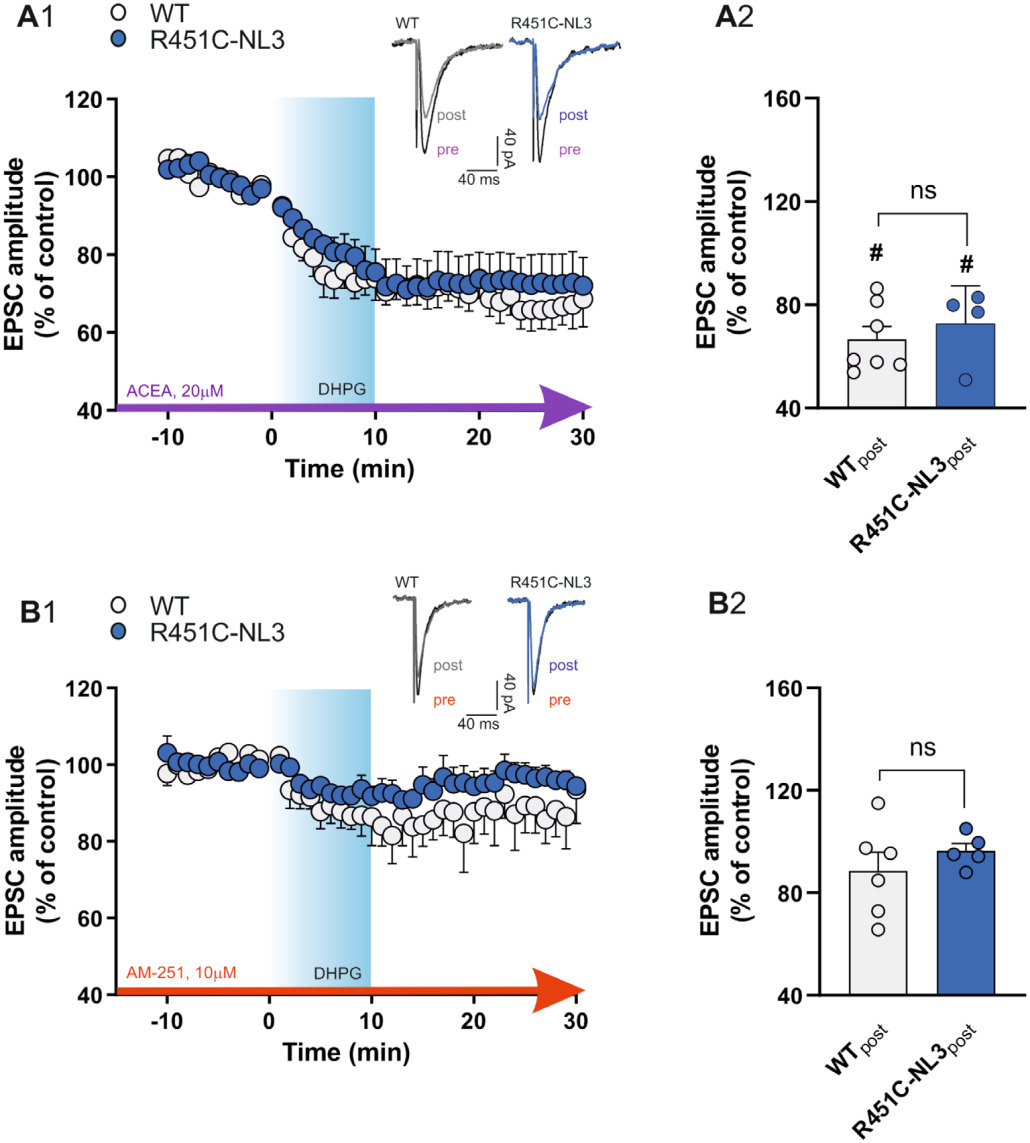
Rescue of DHPG‐induced LTD by exogenous activation of CB1 receptors. (A1) Time course of DHPG‐LTD recorded in the presence of the CB1 receptor agonist ACEA (20 μM) from WT (gray) and R451C‐NL3 (blue) slices. Data are represented as mean ± SEM. (A2) The bar graph reports average EPSC amplitudes measured after DHPG (POST, last 5 min of recording), as a percentage of the mean amplitude at baseline (5 min before DHPG). (B1) Summary plot reporting the time course of mGluI‐LTD in the presence of the CB1 receptor antagonist AM251 (10 μM). Data are represented as mean ± SEM. (B2) The histogram reports the average EPSC amplitudes measured after DHPG (POST, last 5 min of recording), reported as a percentage of the mean amplitude at baseline (5 min before DHPG). Each dot represents a single measurement. Means ± SEM of data are reported. Paired *t* test comparison of PRE versus POST values: #*p* ≤ 0.05. Unpaired *t* test comparison of WT versus R451C‐NL3 POST values: (ns) *p* > 0.05.

We previously showed that the activation of the endocannabinoid (eCB) signaling was able to induce a partial rescue of HFS‐LTD in R451C‐NL3 corticostriatal slices (Martella et al. 2018). We therefore investigated whether the exogenous activation of the eCB system was also able to rescue the pharmacological LTD induced by DHPG application. Corticostriatal slices from mutant and WT mice were preincubated with the highly selective CB1 receptor (CB1R) agonist ACEA (20 μM; Figure S2A). In WT slices, DHPG application in the presence of ACEA induced an LTD (Figure 3A1,A2; *EPSC amplitude post‐DHPG*: WT_POST‐DHPG_ = 66.68 ± 4.96% of pre‐DHPG control, *n* = 7; paired *t*‐test, *t*(6) = 6.892, ***p = 0.0005) of amplitude similar to the DHPG‐LTD recorded in control conditions (Figure 2C2). The observation that ACEA preincubation did not modify the amplitude of DHPG‐induced LTD suggests that mGluI receptor activation is able to induce sufficient CB1R activation to induce this form of corticostriatal LTD in WT slices. In fact, mGluI receptors play a pivotal role in modulating synaptic plasticity at excitatory glutamatergic synapses via different signaling pathways, including eCB production (Anwyl 1999; Robbe et al. 2002; Bellone et al. 2008; Bonsi et al. 2008; Földy et al. 2013; D'Antoni et al. 2014). Conversely, in R451C‐NL3 slices, exogenous CB1R activation exerted an additive effect with respect to DHPG, since preincubation with ACEA was able to fully rescue the expression of a DHPG‐LTD with magnitude comparable to the pharmacological LTD recorded in WT slices (Figure 3A1,A2; *EPSC amplitude post‐DHPG*: R451C‐NL3_POST‐DHPG_ = 72.69% ± 7.35% of pre‐DHPG control, *n* = 4; paired *t*‐test, *t*(3) = 3.716, *p = 0.034; WT_POST‐DHPG_ vs. R451C‐NL3_POST‐DHPG_, unpaired *t*‐test, *t*(9) = 0.702, *p* = 0.500). In line with these observations, DHPG‐LTD expression was prevented by slice preincubation with the CB1R‐specific antagonist AM‐251, and only a mild depression of synaptic transmission was observed in both genotypes (Figure 3B1,B2; *EPSC amplitude post‐DHPG*: WT_POST‐DHPG_ = 88.53% ± 7.31% of pre‐DHPG control, *n* = 6; paired *t*‐test, *t*(5) = 1.556, *p* = 0.180; R451C‐NL3_POST‐DHPG_ = 96.36% ± 2.86% of pre‐DHPG control, *n* = 5; paired *t*‐test, *t*(4) = 1.306, *p* = 0.262; WT_POST‐DHPG_ vs. R451C‐NL3_POST‐DHPG_, unpaired *t*‐test, *t*(9) = 0.923, *p* = 0.380). Overall, these data suggest that in slices from R451C‐NL3 mice, the DHPG‐LTD deficit may depend on the mGluI receptors' inability to induce eCB production. The observations that in R451C‐NL3 slices the mGluI receptor agonist DHPG induces only a transient depression of EPSC amplitude, and that a rescue of pharmacological LTD is obtained with the CB1R agonist ACEA are in support of this hypothesis.

### 3.4 Activation of mGluI Receptors Does Not Potentiate NMDA Receptor‐Mediated Currents in R451C‐NL3 Striatal Slices

**FIGURE 4:**
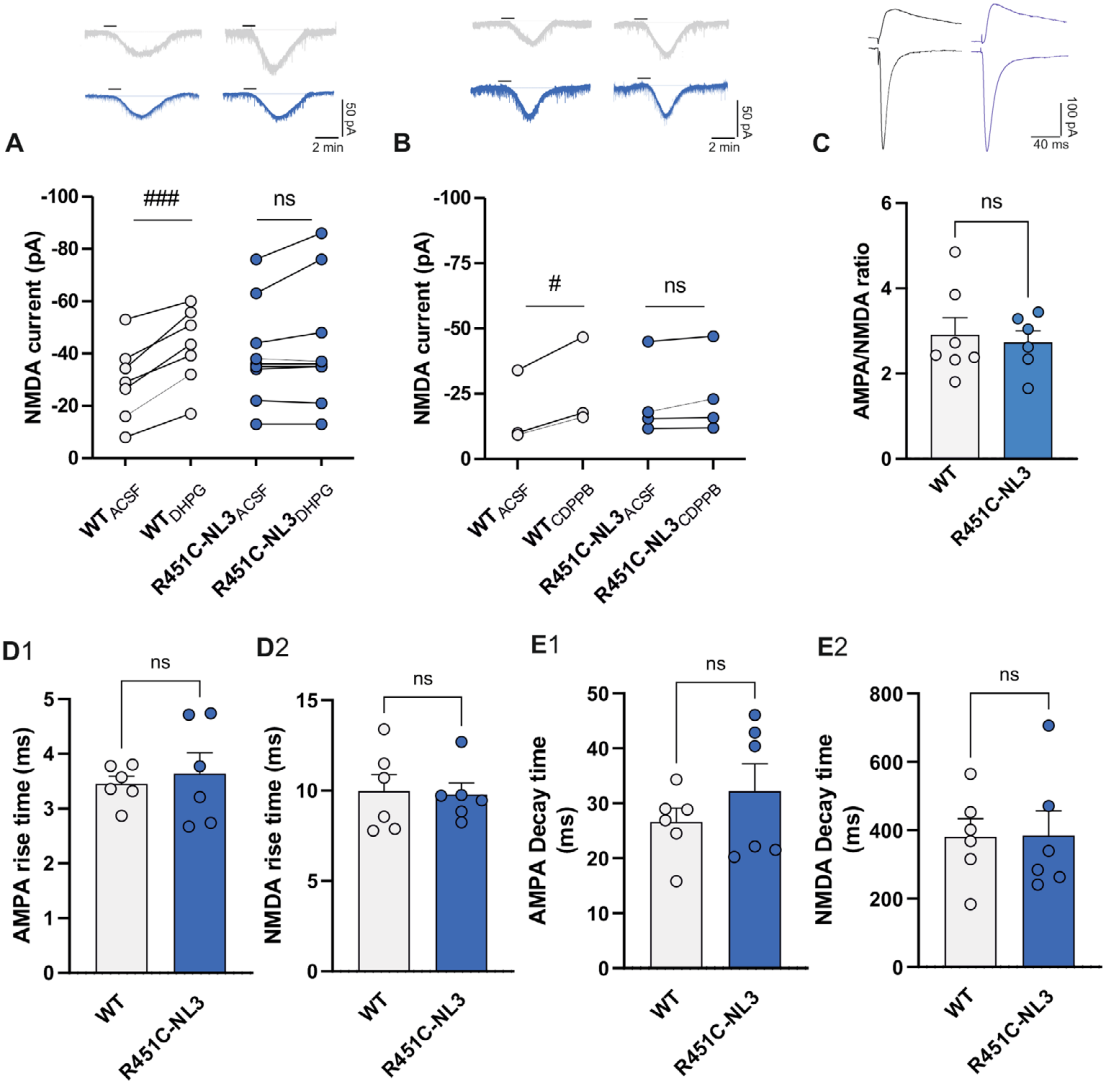
Electrophysiological analysis of AMPA and NMDA receptor‐mediated responses in SPNs from R451C‐NL3 mice. (A) *Top*. Representative traces (WT: gray, R451C‐NL3: blue) showing the NMDA‐mediated current before and after DHPG application. *Bottom*. Before‐after graph reporting the amplitude of the current induced by 30 μM NMDA in WT and R451C‐NL3 SPNs before and after a 5‐min bath application of 50 μM 3,5‐DHPG. Each dot represents a single measurement. Mean ± SEM of data is reported. Paired *t*‐test comparison: (ns) *p* > 0.05; ###*p* = 0.0004. (B) *Top*. Representative traces (WT: gray, R451C‐NL3: blue) showing the NMDA‐mediated current before and after application of the mGlu5 receptor positive allosteric modulator CDPPB. *Bottom*. Before‐after graph reporting the amplitude of the current induced by 30 μM NMDA in WT and R451C‐NL3 SPNs, in ACSF and after bath application of CDPPB (50 μM, 5 min), on the amplitude of the current induced by NMDA. Each dot represents a single measurement. Mean ± SEM of data is reported. Paired *t*‐test comparison: (ns) *p* > 0.05; #*p* ≤ 0.05. (C) *Top*. Representative traces of EPSCs recorded at V_H_ = −70 mV for AMPA receptor‐mediated currents and V_H_ = +40 mV for NMDA receptor‐mediated currents. *Bottom*. Summary plot of the AMPA/NMDA receptor‐mediated current amplitude ratio measured in SPNs from WT and R451C‐NL3 mice. Each dot represents a single measurement. Mean ± SEM of data is reported. Unpaired *t*‐test between WT and R451C‐NL3 mice: (ns) *p* > 0.05. (D) Summary plots of the rise time of (D1) AMPA‐EPSCs (V_H_ = −70 mV), and (D2) NMDA‐EPSCs (V_H_ = +40 mV). Each dot represents a single measurement. Mean ± SEM of data is reported. Unpaired *t*‐test between WT and R451C‐NL3 mice: (ns) *p* > 0.05. (E) Summary plots of the decay time of (E1) AMPA‐EPSCs (V_H_ = −70 mV), and (E2) NMDA‐EPSCs (V_H_ = +40 mV). Each dot represents a single measurement. Mean ± SEM of data is reported. Unpaired *t*‐test between WT and R451C‐NL3 mice: (ns) *p* > 0.05.

As observed in different brain regions, in the dorsal striatum, ionotropic NMDA glutamate receptor‐mediated inward currents/depolarization are enhanced by the simultaneous activation of metabotropic mGlu5 receptors (Pisani et al. 1997, 2001). Hence, to further investigate mGluI receptor function in R451C‐NL3 striatum, we analyzed the potentiation of NMDA receptor‐mediated currents induced by bath application of DHPG during whole‐cell patch‐clamp recordings from SPNs. Repetitive bath applications of NMDA (30 μM, 30 s, 6 min apart) induced a transient inward current of similar amplitude in SPNs from both WT and R451C‐NL3 mice (Figure S2B; WT = −28.90 ± 2.80 pA, *n* = 17; R451C‐NL3 = −29.86 ± 3.55 pA, *n* = 24; Mann–Whitney test, *U* = 200.0, *p* = 0.923). After 5 min of incubation with 50 μM DHPG, a significant enhancement of the NMDA‐mediated inward current was observed in WT SPNs, but not in R451C‐NL3 slices (Figure 4A; WT_ACSF_ = −29.26 ± 5.57 pA, WT_DHPG_ = −42.59 ± 5.61 pA, *n* = 7, paired *t*‐test, *t*(6) = 6.933, ##*p* = 0.0004; R451C‐NL3_ACSF_ = −40.11 ± 6.43 pA, R451C‐NL3_DHPG_ = −43.00 ± 7.96 pA, *n* = 9, paired *t*‐test, *t*(8) = 1.680, *p* = 0.1315). In a further set of experiments, we additionally verified whether an mGlu5 receptor positive allosteric modulator (PAM), CDPPB, was able to potentiate the NMDA receptor‐mediated responses in SPNs from mutant mice. As shown in Figure 4B, similar to DHPG, 50 μM CDPPB increased NMDA receptor‐mediated inward currents in WT slices, whereas it failed to enhance the NMDA‐induced current response in SPNs from R451C‐NL3 mice (Figure 4B; WT_ACSF_ = −17.73 ± 8.14 pA, WT_CDPPB_ = −26.73 ± 9.94 pA, *n* = 3, paired *t*‐test, *t*(2) = 4.959, #*p* = 0.0383; R451C‐NL3_ACSF_ = −22.50 ± 7.61 pA, R451C‐NL3_CDPPB_ = −24.41 ± 7.88 pA, *n* = 4, paired *t*‐test, *t*(3) = 1.734, *p* = 0.1813). These data show that the positive modulation exerted by activation of mGlu5 receptors on NMDA‐mediated responses is disrupted by the R451C‐NL3 mutation. To verify whether this alteration was dependent on a dysfunction of NMDA receptors, we further analyzed the ionotropic glutamate receptor‐mediated postsynaptic currents in SPNs. Our findings did not show significant differences in the AMPA/NMDA ratio between WT and R451C‐NL3 slices (Figure 4C; WT = 2.911 ± 0.401, *n* = 7; R451C‐NL3 = 2.732 ± 0.273, *n* = 6; unpaired *t*‐test, *t*(11) = 0.356, *p* = 0.7290). AMPA‐ and NMDA‐mediated current kinetics were also similar between the two genotypes (Figure 4D1,D2,E1,E2; V_H_ = −70 mV, AMPA rise time: WT = 3.450 ± 0.142 ms, R451C‐NL3 = 3.641 ± 0.370 ms, unpaired *t*‐test, *t*(10) = 0.474, *p* = 0.6458; V_H_ = +40 mV, NMDA rise time: WT = 9.967 ± 0.926 ms, R451C‐NL3 = 9.787 ± 0.627 ms, unpaired *t*‐test, *t*(10) = 0.161, *p* = 0.8752; V_H_ = −70 mV, AMPA decay time: WT = 26.57 ± 2.52 ms, R451C‐NL3 = 32.20 ± 4.94 ms, unpaired *t*‐test, *t*(10) = 1.015, *p* = 0.3342; V_H_ = +40 mV, NMDA decay time: WT = 381.2 ± 52.6 ms, R451C‐NL3 = 384.2 ± 72.7 ms, unpaired *t*‐test, *t*(10) = 0.034, *p* = 0.9737). Overall, these data indicate that the R451C mutation does not affect either the AMPA or the NMDA receptor function, hence suggesting an impairment of mGlu5 receptor function.

### 3.5 R451C‐NL3 Mice Exhibit a Partial Postsynaptic Remodeling in the Dorsal Striatum

**FIGURE 5:**
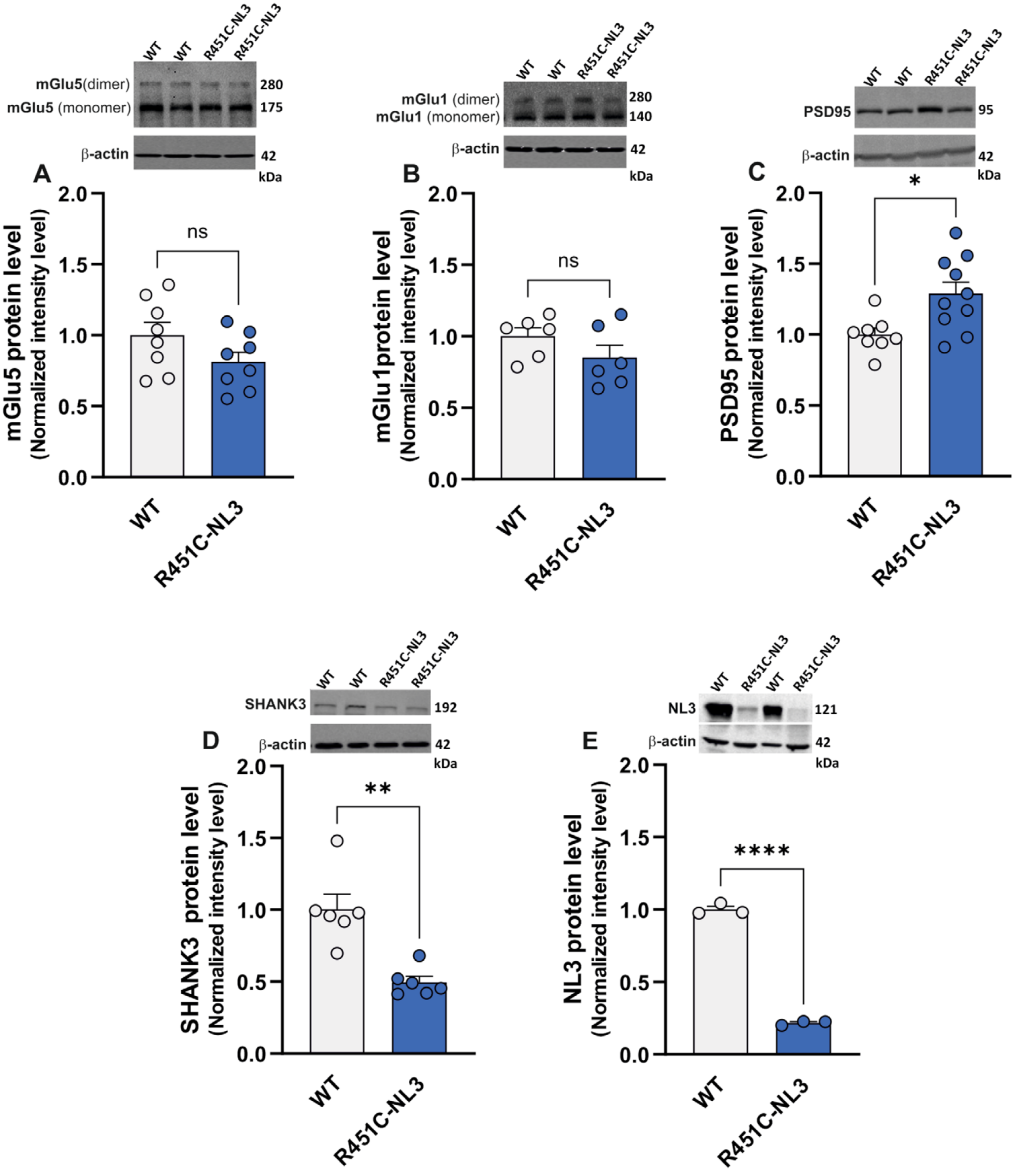
Expression level of postsynaptic proteins in the dorsal striatum of R451C‐NL3 mice: (A) mGlu5 receptor, (B) mGlu1 receptor, (C) PSD95, (D) SHANK3, (D) NL3. *Top*. Representative Western blot quantification of protein levels in dorsal striatum extracts from WT and R451C‐NL3 mice. *Bottom*. The bar graphs show single measurements (dots) and mean ± SEM of the quantification data of the indicated proteins. Unpaired *t*‐test: (ns) *p* > 0.05; *p = 0.011; **p = 0.001; ****p < 0.0001.

In line with accumulating evidence indicating alterations in the function of group I mGlu receptors in diverse brain regions of different ASD experimental models (Auerbach et al. 2011; Baudouin et al. 2012), our findings suggest an impairment of mGluI receptor signaling in the dorsal striatum of R451C‐NL3 mice. To investigate whether this impairment might be attributable to alterations in the protein expression level of striatal mGluI receptors, the protein levels of mGlu5 and mGlu1 receptors were evaluated in total lysates of dorsal striatum from WT and R451C‐NL3 mice. We observed a slight, nonsignificant decrease in both mGlu1 and mGlu5 receptor subtypes (Figure 5A,B; mGlu5: WT = 1.000 ± 0.090, *N* = 8; R451C‐NL3 = 0.812 ± 0.069, *N* = 8; unpaired *t*‐test, *t*(14) = 1.652, *p =* 0.121; mGlu1: WT = 1.000 ± 0.059, *N* = 6; R451C‐NL3 = 0.853 ± 0.086, *N* = 6; unpaired *t*‐test, *t*(10) = 1.404, *p =* 0.191). Group I mGlu5 and mGlu1 receptors are mainly postsynaptic, and both PSD‐95 and SHANK3 play a critical role in organizing and scaffolding signaling molecules at the PSD, influencing receptor trafficking, anchoring, and expression (Iasevoli et al. 2013; Zieger and Choquet 2021). Notably, we observed a significant increase in the protein level of PSD‐95 in the dorsal striatum of R451C‐NL3 mice (Figure 5C; WT = 1.000 ± 0.045, *N* = 8; R451C‐NL3 = 1.288 ± 0.082, *N* = 10; unpaired *t*‐test, *t*(16) = 2.861, *p = 0.011), whereas the protein level of SHANK3 was significantly reduced (Figure 5D; WT = 1.000 ± 0.105, *N* = 6; R451C‐NL3 = 0.497 ± 0.040, *N* = 6; unpaired *t*‐test, *t*(10) = 4.517, **p = 0.001). Notably, our Western blot analysis of dorsal striatum lysates showed a considerable reduction in NL3 protein level (Figure 5E; WT = 1.000 ± 0.222, *N* = 3; R451C‐NL3 = 0.217 ± 0.008, *N* = 3; unpaired *t*‐test, *t*(4) = 32.89, ****p < 0.0001), in line with previous reports from different brain areas of the R451C‐NL3 mouse model (Tabuchi et al. 2007; Trobiani et al. 2018), as well as heterologous expression cellular systems (De Jaco et al. 2010).

Additionally, we examined the protein levels of the AMPA and NMDA ionotropic glutamate receptor subunits. Our analysis suggests a lack of significant differences between the two genotypes in the protein levels of the NMDA receptor subunits GluN2A (Figure S3A; WT = 1.000 ± 0.053, *N* = 3; R451C‐NL3 = 1.155 ± 0.225, *N* = 3; unpaired *t*‐test, *t*(4) = 0.667, *p* = 0.541) and GluN2B (Figure S3B; WT = 1.000 ± 0.084, *N* = 6; R451C‐NL3 = 1.072 ± 0.152, *N* = 5; unpaired *t*‐test, *t*(9) = 0.436, *p* = 0.674). Likewise, no differences were observed in the levels of the AMPA receptor subunits GluA1 (Figure S3C; WT = 1.000 ± 0.105, *N* = 5; R451C‐NL3 = 0.853 ± 0.042, *N* = 6; unpaired *t*‐test, *t*(9) = 1.399, *p* = 0.195) and GluA2 (Figure S3D; WT = 1.000 ± 0.156, *N* = 3; R451C‐NL3 = 1.021 ± 0.062, *N* = 3; Mann–Whitney test, *U* = 3, *p* = 0.700).

Overall, our findings show that the R451C mutation in the NL3 gene impairs the structure and function of the striatal glutamatergic synaptic machinery. Conversely, we did not observe significant changes in the cortical protein levels of either mGlu5 receptor (Figure S4A; WT: 1.000 ± 0.037, *n* = 9, R451C‐NL3: 0.968 ± 0.086, *n* = 7, unpaired *t*‐test, *t*(14) = 0.410, *p* = 0.688) or PSD95 (Figure S4B; WT: 1.000 ± 0.258, *n* = 6, R451C‐NL3: 0.869 ± 0.184, *n* = 5, unpaired *t*‐test, *t*(9) = 0.396, *p* = 0.702).

### 3.6 Altered Expression of mGluI‐Associated Synaptic Proteins in the R451C‐NL3 Striatum

**FIGURE 6:**
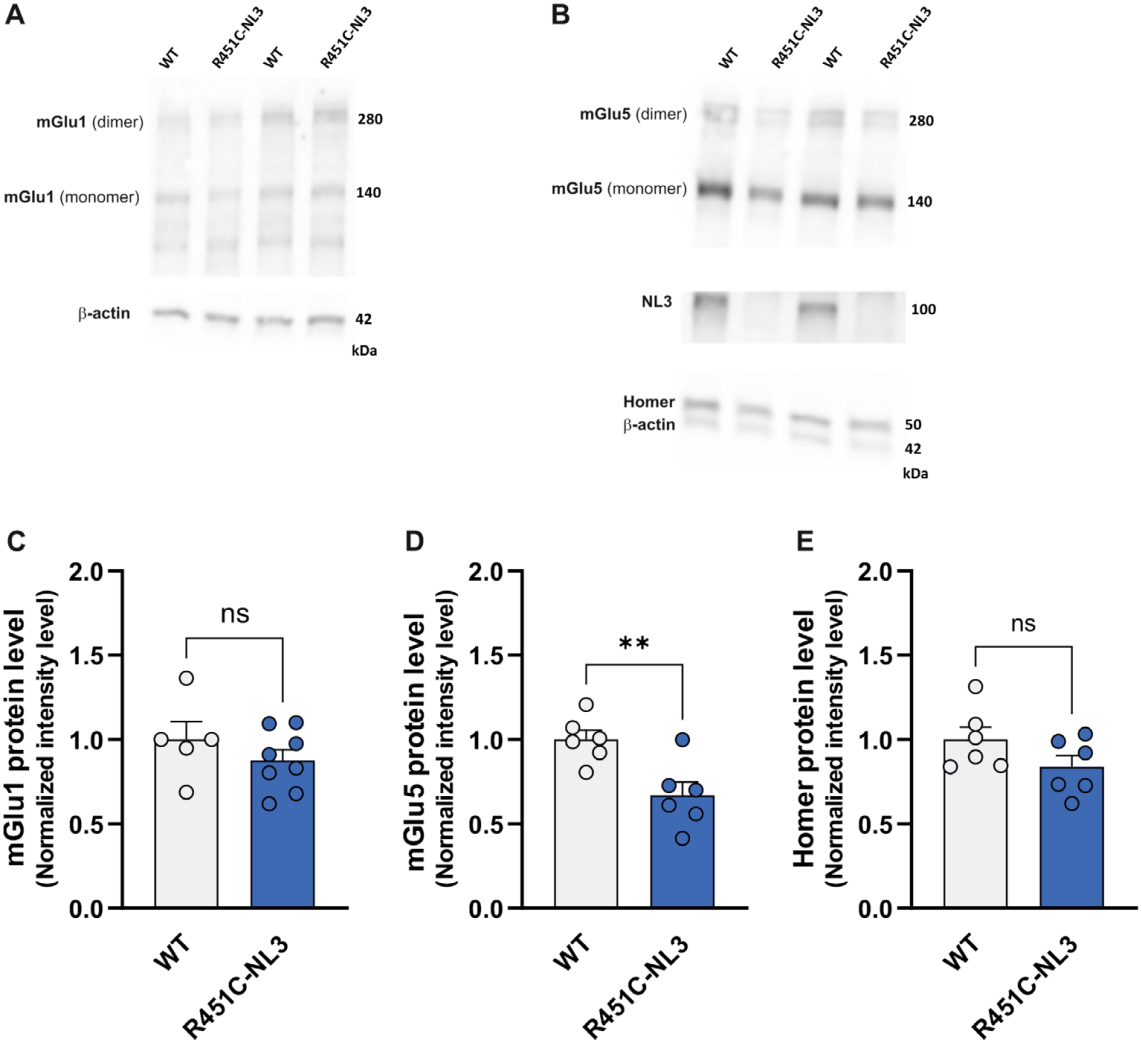
Protein expression level of mGluI receptors in synaptosomal preparations of R451C‐NL3 dorsal striatum. *Top*. Representative Western blot of (A) mGlu1 receptor and (B) mGlu5 receptor, NL3 and Homer protein levels in synaptosomal preparations from R451C‐NL3 and WT dorsal striatum. *Bottom*. The bar graphs show single measurements (dots), and mean ± SEM of (C) mGlu1 receptor, (D) mGlu5 receptor, (E) Homer protein levels. Note that NL3 was undetectable in R451C‐NL3 synaptosomes. Unpaired *t*‐test: (ns) *p* > 0.05; **p ≤ 0.01.

Group I mGlu receptors are mainly localized postsynaptically, in the perisynaptic area where they can be recruited by the high levels of glutamate released during sustained synaptic transmission, contributing to long‐term plastic changes (Lujan et al. 1996; Paquet and Smith 2003; Wierońska and Pilc 2009; D'Antoni et al. 2014). We analyzed their specific expression in synaptosomal preparations obtained from the dorsal striatum of both R451C‐NL3 and WT mice. Synaptic mGlu1 receptor levels were not statistically different between R451C‐NL3 and control littermates (Figure 6A; WT: 1.000 ± 0.108, *n* = 5, R451C‐NL3: 0.877 ± 0.063, *n* = 8, unpaired *t*‐test, *t*(11) = 1.065, *p* = 0.310). Conversely, Western blot experiments showed a statistically significant decrease in mGlu5 receptor protein levels in R451C‐NL3 striatal synaptosomes with respect to WT (Figure 6B; WT: 1.000 ± 0.055, *n* = 6, R451C‐NL3: 0.669 ± 0.080, *n* = 6, unpaired *t*‐test, *t*(10) = 3.399, **p = 0.007). The synaptic scaffolding protein Homer is a known binding partner of group I mGlu receptors (Bockaert et al. 2021; Mao et al. 2022), whose expression is required in PSD remodeling, modulation of glutamate receptor‐mediated functions, regulation of calcium signaling, and plasticity of excitatory synapses (Clifton et al. 2019; Mao et al. 2022). We therefore evaluated whether the reduction in mGlu5 receptor synaptic expression was associated with changes in Homer protein levels. We found no significant difference in Homer protein level between R451C‐NL3 and WT synaptosomal preparations (Figure 6C; WT = 1.000 ± 0.075, *N* = 6; R451C‐NL3 = 0.838 ± 0.068, *N* = 6; unpaired *t*‐test, *t*(10) = 1.614, *p =* 0.138). In different ASD preclinical models, evidence of mGluI receptor dysfunction has been provided in the cortex (Vicidomini et al. 2017). We therefore analyzed the receptor protein levels in cortical synaptosomal preparations from R451C‐NL3 and WT mice. Similar to the striatum, also in cortical synaptosomes we observed a significant reduction in mGlu5 receptor level, whereas mGlu1 receptor and Homer levels were not significantly altered (Figure S4C–E; mGlu1, WT: 1.000 ± 0.047, *n* = 8, R451C‐NL3: 0.865 ± 0.045, *n* = 10, unpaired *t*‐test, *t*(16) = 2.048, *p* = 0.057; mGlu5, WT = 1.000 ± 0.083, *N* = 5; R451C‐NL3 = 0.696 ± 0.050, *N* = 5; unpaired *t*‐test, *t*(8) = 3.142, *p = 0.014; Homer, WT = 1.000 ± 0.058 *N* = 5; R451C‐NL3 = 0.881 ± 0.076, *N* = 5; unpaired *t*‐test, *t*(8) = 1.246, *p =* 0.248).

Overall, our findings support a selective impairment of mGlu5 receptor in R451C‐NL3 mice, suggesting that its dysfunction might underscore the deficits of synaptic plasticity that we observed in the dorsal striatum.

## 4 Discussion

**FIGURE 7:**
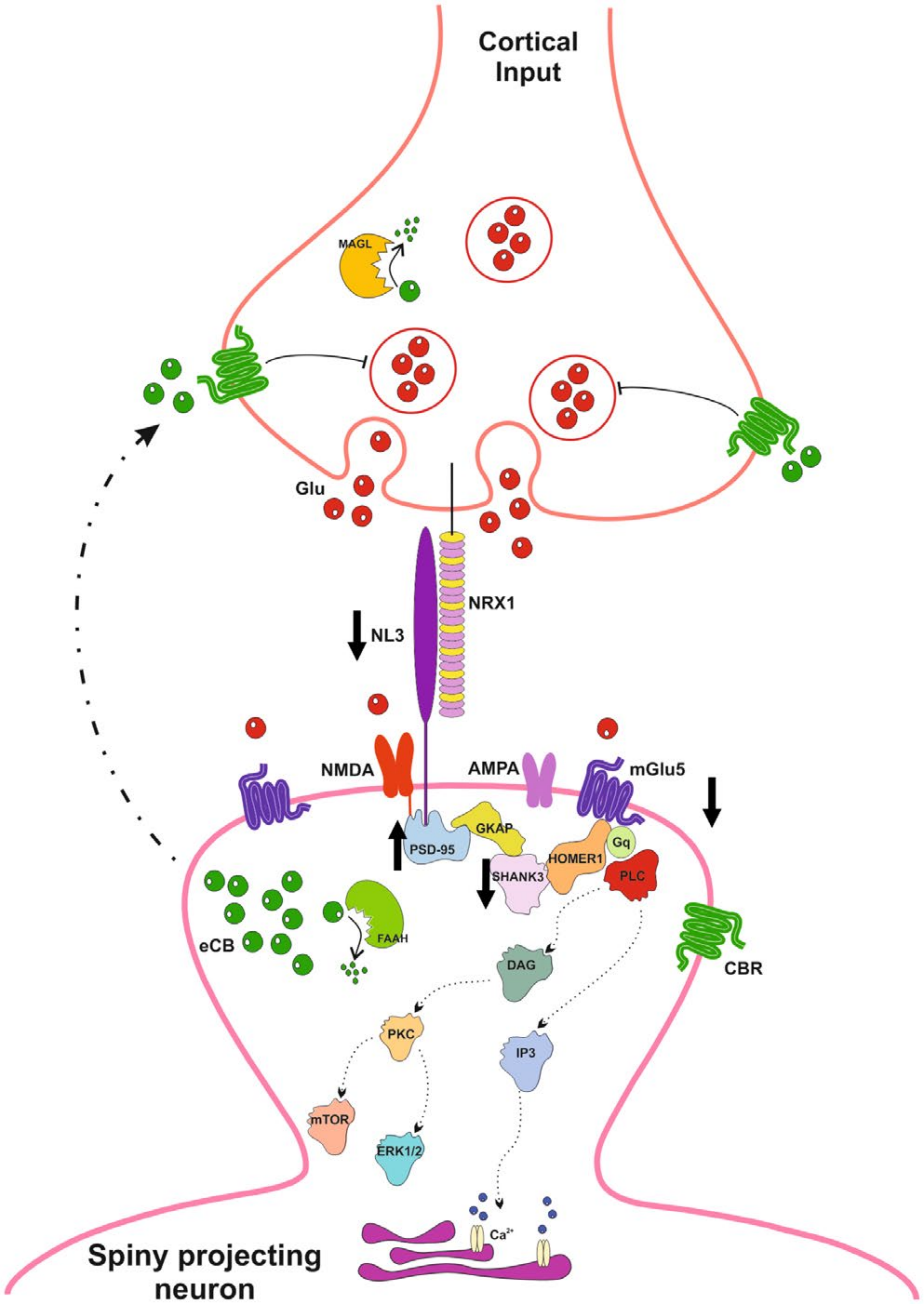
Molecular pathways implicated in corticostriatal synaptic function. The NL3 protein interacts with the ionotropic NMDA receptor–PSD95 complex, which is linked to SHANK3 scaffolding protein and Homer through guanylate kinase‐associated protein (GKAP). These proteins, through their coiled‐coil domains, create multimeric structures bridging SHANKs to mGlu5 receptors and IP3. The binding of glutamate to the mGlu5 receptor triggers the activation of the Gq protein signaling pathways, activating phospholipase C (PLC), which catalyzes the breakdown of phosphatidylinositol‐4,5‐bisphosphate (PIP2) into 1,2‐Diacylglycerol (DAG) and IP3, acting as second messengers. DAG stimulates protein kinase C (PKC), initiating a series of intracellular signal transduction pathways. IP3 engages with receptors located on the endoplasmic reticulum (ER), triggering the liberation of Ca^2+^ ions into the cytoplasm and activating Ca^2+^ concentration‐dependent signaling mechanisms. The endocannabinoid retrograde signaling and the hydrolysis by the degradative enzymes FAAH and MAGL are also included in the cartoon. Thick arrows indicate changes in dorsal striatum synaptic protein levels reported in the present work. CBR, cannabinoid receptors; Glu, glutamate; mGluI, group I metabotropic glutamate receptors; NRXN1, neurexin 1.

Neurodevelopmental disorders encompass heterogeneous phenotypes; however, genetic studies have shown that these different conditions share a significant enrichment of associated/candidate genes located at the pre‐ or postsynaptic compartments or known to directly regulate synaptic functions in neurons (Meredith 2015). These observations are supported by anatomical data obtained from both post‐mortem tissue and animal models, showing an altered morphology of dendritic spines and filopodia across different brain regions, leading to the use of the term ‘synaptopathies’ to describe a multitude of conditions, including ASD, directly affecting synaptic processing and plasticity (Brose et al. 2010). Despite evidence of synaptic defects as a substrate of ASD, the underlying molecular mechanisms have only partially been uncovered (Delorme et al. 2013; Meredith 2015; Trobiani et al. 2020; Bonsi et al. 2022). Synapse formation is a tightly regulated process, involving the gathering of specific cell adhesion molecules and scaffolding proteins, which significantly influence whether an initial contact evolves into an excitatory or an inhibitory synapse (Craig and Boudin 2001; Graf et al. 2004; Craig and Kang 2007). Neuroligins play a significant role in defining synaptic properties (Varoqueaux et al. 2006), and their impairment can alter the arrangement of neurotransmitter receptors (Chanda et al. 2017). In particular, the R451C substitution in the NL3 gene has been implicated in the dysfunction of both inhibitory and excitatory synaptic transmission (Trobiani et al. 2020). Indeed, in layer 2/3 of the somatosensory cortex of the R451C‐NL3 mouse model, an increased frequency of spontaneous inhibitory events and impaired eCB‐mediated signaling have been demonstrated (Tabuchi et al. 2007; Speed et al. 2015). In the same mouse model, an increase of AMPA and NMDA receptor‐mediated excitatory synaptic transmission in the CA1 area of the hippocampus (Etherton, Tabuchi, et al. 2011), and an imbalance in the excitatory/inhibitory postsynaptic activity in pyramidal cells of the basolateral amygdala (Hosie et al. 2018) were reported, together with an impairment of NMDA receptor activity in the medial prefrontal cortex (Cao et al. 2022). In the ventral striatum/nucleus accumbens, the R451C‐NL3 mutation impairs synaptic inhibitory transmission onto striatonigral SPNs but does not affect the expression of DHPG‐LTD (Rothwell et al. 2014). Conversely, we previously showed that HFS‐LTD expression is impaired at corticostriatal glutamatergic synapses of the dorsal striatum (Martella et al. 2018). Altogether, these observations suggest that the R451C‐NL3 mutation produces region‐specific effects within the brain, and the striatum in particular. Previous work implicated striatal abnormalities as the basis of the social deficits and the restricted and repetitive behaviors characteristic of ASD (Braden et al. 2017; Balsters et al. 2018; Gotts et al. 2019; Hegarty II et al. 2020). In addition to the two major phenotypes supporting an ASD diagnosis, autistic children may also face difficulties in movement planning during locomotion, further pointing to the striatum as an anatomical substrate of ASD‐associated phenotypes (Vernazza‐Martin et al. 2005). Indeed, the striatum is anatomically and functionally organized in a ventral part, mediating limbic functions of motivation, reward, and emotion‐related behaviors, and a dorsal region, implicated in cognitive and motor functions, such as sensorimotor processing, goal‐directed, and automated behavior (Chen et al. 2015). Such functional segregation relies on the topographical organization of the glutamatergic inputs to the striatum, which are distributed in a dorsomedial‐to‐ventrolateral arrangement from the sensorimotor, associative, and limbic cortices (Voorn et al. 2004), reflecting the involvement of this brain area in the integration of sensorimotor, cognitive, and motivational/emotional information, which is the basis for the expression of the motor, sensory, cognitive, and social behaviors affected in ASD (Balleine et al. 2007; Ecker et al. 2015; Horder et al. 2018; Pote et al. 2019). In particular, the dorsal striatum represents a key brain region for the initiation and control of movements, as well as for habitual and repetitive actions (Costa et al. 2004; Yin and Knowlton 2006; Yin et al. 2006, 2009; Yin 2010; Kohls et al. 2014; Burton et al. 2024; Hart, Burton, and Balleine 2024; Hart, Burton, Nolan, and Balleine 2024). Despite this evidence, while there is a substantial amount of work on the role of the ventral striatum in ASD, the dorsal region has so far been poorly investigated. Notably, imaging studies have revealed anatomical differences in the striatum of individuals with ASD (Hollander et al. 2005; Rojas et al. 2006; Evans et al. 2024). Accordingly, striatal neurophysiological alterations and neuroanatomical changes have been reported in ASD animal models (Trobiani et al. 2020; Thabault et al. 2022). In particular, the proposed dorso‐ventral anatomical subdivision (Fuccillo 2016) attributes the generation of stereotypies and repetitive behaviors to the dorsal striatum (Burton et al. 2024). Our behavioral analysis of the R451C‐NL3 mice, consistent with previous findings, indicates impairments across all the domains relevant to ASD (Tabuchi et al. 2007; Etherton, Földy, et al. 2011; Rothwell et al. 2014; Burrows et al. 2015). Notably, we reported alterations in the marble test, for the first time in the R451C‐NL3 mouse model, indicating stereotypic behavior. Our functional and molecular analyses, in line with the behavioral observations, indicate significant alterations in the dorsal striatum. Notably, we observed defects in the expression of diverse forms of long‐term corticostriatal synaptic plasticity. Indeed, as we previously reported, HFS‐LTD is impaired in the dorsal striatum of R451C‐NL3 mice (Martella et al. 2018). Interestingly, the delivery of the LTP induction protocol was not able to induce a significant change in corticostriatal synaptic strength, therefore supporting an impairment of both forms of activity‐dependent long‐term plasticity. Despite the signaling pathways involved in corticostriatal LTD and LTP expression have not been completely clarified yet, both are believed to involve postsynaptic mechanisms, including SPN membrane depolarization, mGluI receptors activation, and intracellular calcium increase. Notably, LTD induction is prevented by blockade of mGluI receptors, while it is favored by the activation of these receptors combined with postsynaptic depolarization (Gubellini et al. 2001; Sung et al. 2001; Lovinger 2010). Similarly, it has been previously shown that mGluI receptors are involved in corticostriatal LTP (Gubellini et al. 2003; Neyman and Manahan‐Vaughan 2008; Kroker et al. 2011). Additionally, LTP requires activation of NMDA, muscarinic M1, and either D1 or A2A receptors, insertion of AMPA receptors at the synapse, and phosphorylation of DARPP‐32 (Lovinger 2010), while LTD involves activation of eCB synthesis and release, and activation of presynaptic CB1 receptors, which produces a long‐lasting decrease in glutamate release probability (Lovinger 2010). Similarly to HFS‐LTD, striatal mGluI‐LTD depends on the eCB‐mediated inhibition of glutamate release (Mango and Ledonne 2023), internalization of ionotropic glutamate receptors, and modifications of AMPA receptor subunit composition (Kreitzer and Malenka 2005, 2008). Notably, the present work shows that, in addition to activity‐dependent synaptic plasticity, pharmacological LTD is also impaired in the dorsal striatum of R451C‐NL3 mice, further pointing to an involvement of mGluI receptors in the inability of corticostriatal synapses to undergo long‐lasting changes. Indeed, we showed that DHPG application induces only a transient depression of excitatory synaptic transmission, suggesting dysfunction of the signaling pathway activated by mGluI receptors, which is necessary for DHPG‐LTD. Furthermore, our observation that DHPG does not potentiate NMDA currents in ASD mice suggests that also the impairment of LTP may depend on mGluI receptor dysfunction, as observed in other brain regions (Xiang et al. 2019). A potential mechanism by which mGluI receptors impairment may affect the expression of corticostriatal LTD resides in the ability of these metabotropic receptors to activate eCB signaling. In support of this hypothesis, we show that exogenous activation of CB1 receptors successfully restores the expression of DHPG‐LTD at R451C‐NL3 corticostriatal synapses. On the other hand, CB1 receptor inhibition produces a partial disruption of LTD induced by mGluI activation at WT synapses. These findings suggest that dysfunction of mGluI receptors in the dorsal striatum of R451C‐NL3 mice may prevent retrograde eCB signaling, which is essential for LTD expression (Sergeeva et al. 2007). mGluI receptors functionally and physically interact with ionotropic NMDA receptors at the postsynaptic area (Awad et al. 2000; Benquet et al. 2002; Li et al. 2018; Mao et al. 2022). Previous studies provided evidence for a facilitatory role of mGluI receptors on ionotropic NMDA receptor function in striatal neurons (Pisani et al. 1997, 2001). Interestingly, we now report that activation of mGlu5 receptors, with either the agonist DHPG or the PAM CDPPB, failed to potentiate NMDA receptor‐mediated currents in the R451C‐NL3 dorsal striatum. Remarkably, our research did not uncover alterations in NMDA receptors, as we did not observe alterations in either receptor‐mediated current amplitude or kinetics, or in the AMPA/NMDA ratio. Furthermore, though further work is required, preliminary observations suggest the lack of changes in the protein expression levels of the ionotropic glutamate receptor subunits. These data further point to metabotropic, rather than ionotropic, glutamate receptor dysfunction in the striatum of the R451C‐NL3 mouse model. Indeed, Western blot data demonstrate a significant reduction of the mGlu5 receptor protein level in striatal synaptosomal preparations from R451C‐NL3 mice, characterized by a highly significant reduction of NL3 levels. Notably, our Western blot analysis also disclosed alterations in the expression of other ASD‐relevant postsynaptic proteins, specifically a decrease in SHANK3 and an increase in PSD‐95 striatal levels. PSD‐95 is a major regulator of synaptic maturation that stabilizes NMDA and AMPA receptors on the postsynaptic membrane (Xu et al. 2008; Coley and Gao 2018). Overwhelming evidence correlates PSD‐95 disruption to cognitive and learning deficits in ASD (Kaizuka and Takumi 2018). In animal studies, PSD‐95 deficiency was associated with alterations in the composition and function of NMDA and AMPA receptors, while increased expression led to redistribution of NL2 from inhibitory to excitatory synapses in neuronal cell cultures (Prange et al. 2004; Levinson and El‐Husseini 2005). SHANK3 is a scaffolding protein that can interact with both NLs and Neurexins to coordinate signaling between pre‐ and postsynaptic regions in excitatory glutamatergic synapses (Arons et al. 2012). Specifically, it interacts directly with the GKAP protein, which in turn is associated with the NMDAR‐PSD95 complex, and, on the other hand, with Homer proteins, by forming multimers through the interaction of their coiled‐coil domain and linking to mGluRI and inositol triphosphate (IP3) (Jung and Park 2022). In addition, SHANK proteins bind Homer and PSD‐95 complexes in the postsynaptic density, regulating trafficking and signaling efficiency of mGluRI receptors (Naisbitt et al. 1999; Tu et al. 1999; Piers et al. 2012) (Figure 7).

Notably, increasing evidence supports the involvement in ASD of mGluI receptors, particularly the mGlu5 subtype. Several ASD‐associated genes encode mGlu5 receptor‐interacting proteins, including SHANK3 and Homer scaffold proteins (Arons et al. 2012; Won et al. 2012; Jiang and Ehlers 2013). The expression of mGluI receptors was upregulated at forebrain synapses of infant rats exposed to valproic acid (D'Antoni et al. 2023). mGlu5 receptor‐mediated PI hydrolysis was blunted in the cerebral cortex, hippocampus, and corpus striatum of Ube3am−/*p* + and *Fmr1* knockout mice, modeling Angelman and Fragile X syndromes, respectively (Di Menna et al. 2023). Dysregulation of mGlu5 receptor signaling has been reported in the hippocampus of Fmr1 knockout mice (Huber et al. 2002; Auerbach et al. 2011; Di Menna et al., 2023), whereas tuberous sclerosis mouse models exhibited an impaired mGluI‐dependent LTD (Chévere‐Torres et al. 2012).

In conclusion, the present work reports an overall disruption of the glutamatergic postsynaptic density in the dorsal striatum of the R451C‐NL3 mouse model of ASD, associated with the impairment of bidirectional corticostriatal long‐term synaptic plasticity. Our data point to a blunted mGluI receptors signaling as the culprit of synaptic plasticity dysfunction, causing impaired NMDA receptor‐mediated current enhancement and likely a reduced eCB production. Therefore, our work adds to previous evidence on different experimental models of ASD, implicating disrupted mGluI receptor signaling in the pathophysiology of ASD, albeit with divergent outcomes in the various brain areas and/or experimental models investigated (Trobiani et al. 2020). Accordingly, these receptors have been investigated as possible therapeutic targets in ASD. In accordance with our data, studies reported a rescuing effect of mGlu5 receptor agonists on synaptic plasticity. In particular, SHANK3 knockout mice, similar to our data, showed a mislocalization of mGlu5 receptors at the striatal postsynaptic density, causing an impairment of LTD that was rescued by CDPPB (Wang et al. 2016). Surprisingly, the positive allosteric modulator did not ameliorate the behavioral alterations observed in this ASD mouse model; rather, the mGlu5 receptor antagonist MPEP reversed the hypoactivity in the open field and the increased self‐grooming of SHANK3 mice (Wang et al. 2016). This observation, together with other reports describing beneficial effects of mGlu5 receptor antagonists on the behavioral alterations observed in the BTBR, valproic acid, and *Fmr1* knockout mouse models of ASD (Burket et al. 2011; Michalon et al. 2012; Zoicas and Kornhuber 2019), suggests that the systemic modulation of mGlu5 receptor activity may result in outcomes different from its striatal‐specific activation, and that further studies are needed to dissect the specific role of mGluI receptors in ASD.

## Author Contributions

**Maria Meringolo:** conceptualization, investigation, methodology, writing – original draft, writing – review and editing, data curation, formal analysis, project administration, validation, visualization. **Martina Montanari:** methodology, investigation, writing – original draft, data curation, formal analysis. **Simona D'Antoni:** methodology, investigation, writing – original draft, writing – review and editing, data curation, formal analysis, visualization, validation. **Giuseppina Martella:** investigation, data curation. **Ilham El Atiallah:** investigation, data curation. **Giulia Ponterio:** investigation, data curation. **Annalisa Tassone:** investigation, data curation. **Ingrid Reverte:** methodology, validation. **Daniele Caprioli:** validation, supervision. **Georgios Strimpakos:** methodology, resources. **Luisa Pieroni:** conceptualization, investigation, writing – original draft, methodology, validation, data curation, formal analysis, visualization, supervision, resources. **Maria Vincenza Catania:** writing – review and editing, supervision, validation, conceptualization, funding acquisition. **Paola Bonsi:** conceptualization, funding acquisition, writing – original draft, writing – review and editing, supervision, project administration, validation.

## Conflicts of Interest

The authors declare no conflicts of interest.

## Supporting information


**Appendix S1:** jnc70253‐sup‐0001‐AppendixS1.pdf.

## Data Availability

The data that support the findings of this study are available from the corresponding author upon reasonable request.
