# Supplementary material for: Impairment of Group I Metabotropic Glutamate Receptors in the Dorsal Striatum of the R451C‐Neuroligin 3 Mouse Model of Autism Spectrum Disorder
Source: J Neurochem. 2025 Oct 13;169(10):e70253. doi: 10.1111/jnc.70253 (PMC12516673; doi:10.1111/jnc.70253)

SUPPLEMENTARY INFORMATION

Impairment of group I metabotropic glutamate receptors in the dorsal striatum of the R451C-neuroligin 3 mouse model of Autism Spectrum Disorder

Maria Meringolo[1,2], Martina Montanari[1], Simona D'Antoni[3], Giuseppina Martella[1], Ilham El Atiallah[1], Giulia Ponterio[1], Annalisa Tassone[1], Ingrid Reverte[4], Daniele Caprioli[4], Georgios Strimpakos[5], Luisa Pieroni[2,6], Maria Vincenza Catania[3], Paola Bonsi[1*]

[1]Laboratory of Neurophysiology and Plasticity, IRCCS Fondazione Santa Lucia, Rome, Italy;
[2]Saint Camillus International University of Health and Medical Sciences, Rome, Italy;
[3]Institute for Biomedical Research and Innovation, National Research Council, Catania, Italy;
[4]Department of Physiology and Pharmacology, Sapienza University, and Laboratory of Behavioral Neuropharmacology, IRCCS Fondazione Santa Lucia, Rome, Italy;
[5]Institute of Biochemistry and Cell Biology, National Research Council, Monterotondo, Italy;
[6]Proteomics and Metabolomics Unit, IRCCS Fondazione Santa Lucia, Rome, Italy

[*]Correspondence
Paola Bonsi, Laboratory of Neurophysiology and Plasticity, IRCCS Fondazione Santa Lucia, Rome, Italy.
Email: p.bonsi@hsantalucia.it

Impairment of group I metabotropic glutamate receptors in the dorsal striatum of the R451C-neuroligin 3 mouse model of Autism Spectrum Disorder

Maria Meringolo[1,2], Martina Montanari[1], Simona D'Antoni[3], Giuseppina Martella[1], Ilham El Atiallah[1], Giulia Ponterio[1], Annalisa Tassone[1], Ingrid Reverte[4], Daniele Caprioli[4], Georgios Strimpakos[5], Luisa Pieroni[2,6], Maria Vincenza Catania[3], Paola Bonsi[1*]

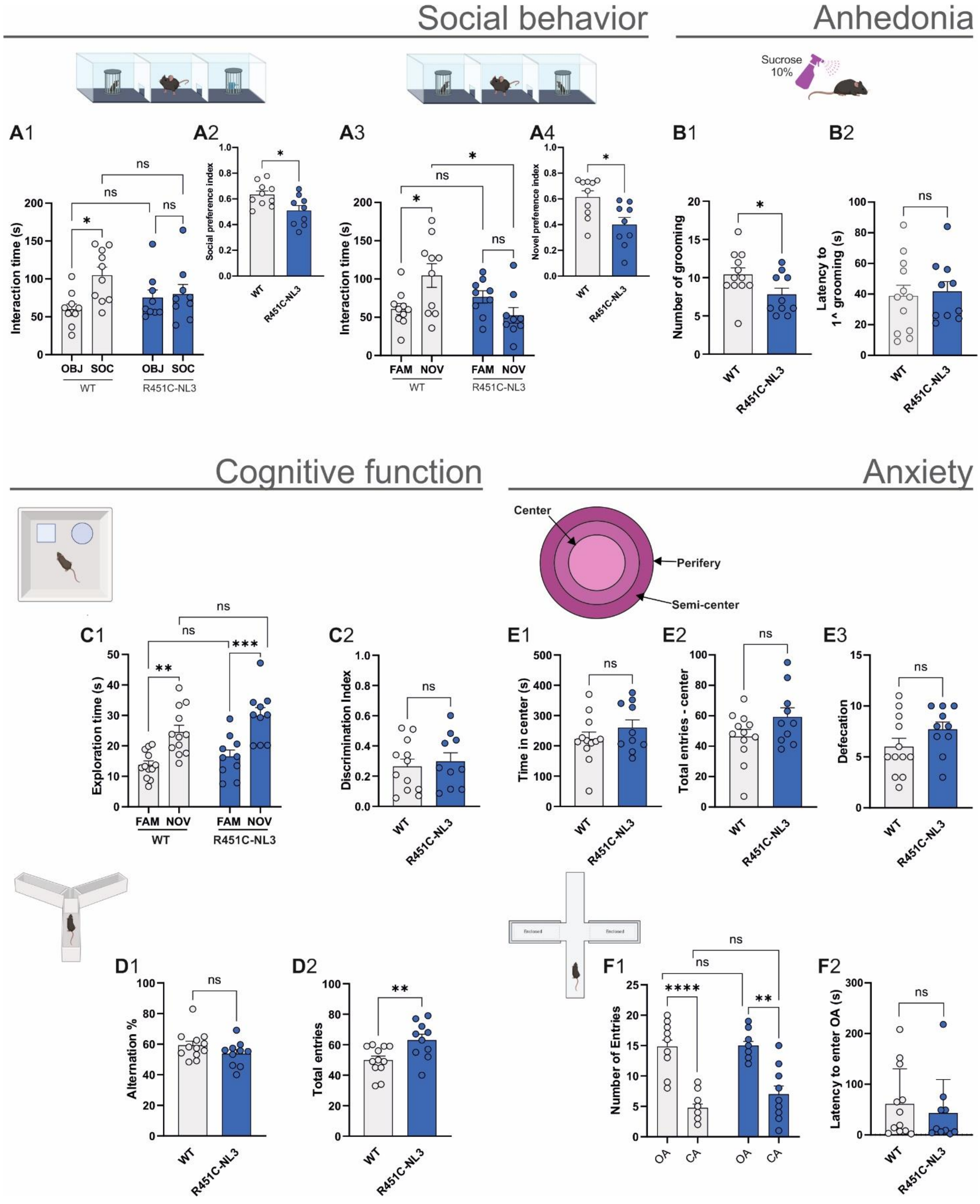

Supplementary Figure 1. Social behaviors: (Top) Cartoon of the 3-chamber apparatus in the two phases of the test. A1) Amount of time spent interacting with the social stimulus (SOC) or non-social

stimulus (OBJ) in WT versus R451C-NL3 mice (Interaction time: WT_{object}= 60.08 ± 6.811s, WT_{social}= 104.8 ± 10.81s, N= 10, Sidak's multiple comparisons test *p = 0.018; R451C-$NL3_{object}$= 75.20 ± 10.16s; R451C-$NL3_{social}$= 80.11 ± 12.42s, N= 9, Sidak's multiple comparisons test p = 0.9997; two-way ANOVA, $F(1,34)_{interaction}$ = 5.978, P = 0.448). A2) Bar graph of the social preference index of control and R451C-NL3 mice ($WT_{social\ preference\ index}$= 0.633 ± 0.028, R451C-$NL3_{social\ preference\ index}$= 0.509 ± 0.038; unpaired t-test, t(17) = 2.607, *p = 0.018). A3) Amount of time spent interacting with the novel stimulus (NOV) or the familiar stimulus (FAM) in the novelty phase of the test (Interaction time: $WT_{familiar}$ = 60.79 ± 7.317s, WT_{novel}= 104.5 ± 15.46s, N= 10, Sidak's multiple comparisons test, *p = 0.037; R451C-$NL3_{familiar}$= 76.54 ± 8.005s; R451C-$NL3_{novel}$= 52.41 ± 10.12s, N= 9, Sidak's multiple comparisons test, p = 0.586; two-way ANOVA, $F(1,34)_{interaction}$ = 9.670, **p = 0.0038). A4) Bar graph of the novel preference index of WT and R451C-NL3 mice ($WT_{novel\ preference\ index}$= 0.615 ± 0.049, R451C-$NL3_{novel\ preference\ index}$= 0.400 ± 0.056; Mann-Whitney test, U = 16, *p = 0.017). Anhedonia: (Top) Cartoon of the sucrose splash test. B1) Number of dorsal self-grooming (WT= 10.42 ± 0.857, N= 12; R451C-NL3= 7.8 ± 0.841, N= 10; unpaired t-test, t(20) = 2.157, *p = 0.043) and (B2) latency to the first grooming of R451C-NL3 mice and their WT littermates (WT= 38.75 ± 7.072 s, N= 12; R451C-NL3= 41.70 ± 6.414 s, N= 10; unpaired t-test, t(20) = 0.304, p = 0.765). <u>Cognitive function:</u> C) Novel object recognition test (NORT). (C1) Summary plots depicting the amount of time spent interacting with the familiar (FAM) or the novel (NOV) object during the test session ($WT_{familiar}$= 13.78 ± 1.286 s, WT_{novel}= 24.56 ± 2.235 s, N= 12; Sidak's multiple comparisons test, **p = 0.003; R451C-$NL3_{familiar}$= 16.54 ± 2.124 s; R451C-$NL3_{novel}$= 29.91 ± 2.660 s, N= 10; Sidak's multiple comparisons test, ***p = 0.0006; one-way ANOVA, F(3,40) = 12.29, ****p < 0.0001). (C2) Bar chart reporting the discrimination index for the novel object (WT= 0.265 ± 0.048, N= 12; R451C-NL3= 0.298 ± 0.056, N= 10; unpaired t-test, t(20) = 0.453, p = 0.655). D) Y maze test. The summary plots report (D1) the percentage of spontaneous alternations (WT= 59.32 ± 2.666 %, N= 12; R451C-NL3= 53.80 ± 2.613 %, N= 10; unpaired t-test, t(20) = 1.464, p = 0.159), and (D2) the total number of entries in the three arms of the Y maze apparatus (WT= 49.83 ± 2.719, N= 12; R451C-NL3= 63.00 ± 3.836, N= 10; unpaired t-test, t(20) = 2.868, **p = 0.0095). <u>Anxiety behaviors:</u> E) Open field test. (E1) Bar charts reporting the time spent in the center of the arena (WT= 222.7 ± 22.92 s, N= 12; R451C-NL3= 260.8 ± 25.21 s, N= 10; unpaired t-test, t(20) = 1.119, p = 0.276), (E2) number of entries in the center zone (WT= 46.25 ± 4.799, N= 12; R451C-NL3= 59.20 ± 5.974, N= 10; unpaired t-test, t(20) = 1.711, p = 0.103) and (E3) number of defecations during the session (WT= 6.0 ± 0.835, N= 12; R451C-NL3= 7.7 ± 0.731, N= 10; unpaired t-test, t(20) = 1.500, p = 0.149). F) Elevated plus maze (EPM). (F1) Summary plots comparing the number of entries of mice in the open

(OA) *vs* closed arms (CA) (WT: open arms= 14.83 ± 1.079, closed arms= 4.750 ± 0.641, N= 12; Sidak's multiple comparisons test, ****p < 0.0001; R451C-NL3: open arms= 15.00 ± 0.683, closed arms= 7.0 ± 1.358, N= 10; Sidak's multiple comparisons test, ****p = 0.0001; one-way ANOVA F(3,40) = 30.33, ****p < 0.0001). (F2) Latency to enter the OA (WT= 61.0 ± 20.04 s, N= 12; R451C-NL3= 43 ± 21.02 s, N= 10; Mann-Whitney test, U = 49.50, p = 0.507). Each dot represents a single measurement. Mean ± SEM of data is reported.

Impairment of group I metabotropic glutamate receptors in the dorsal striatum of the R451C-neuroligin 3 mouse model of Autism Spectrum Disorder

Maria Meringolo[1,2], Martina Montanari[1], Simona D'Antoni[3], Giuseppina Martella[1], Ilham El Atiallah[1], Giulia Ponterio[1], Annalisa Tassone[1], Ingrid Reverte[4], Daniele Caprioli[4], Georgios Strimpakos[5], Luisa Pieroni[2,6], Maria Vincenza Catania[3], Paola Bonsi[1*]

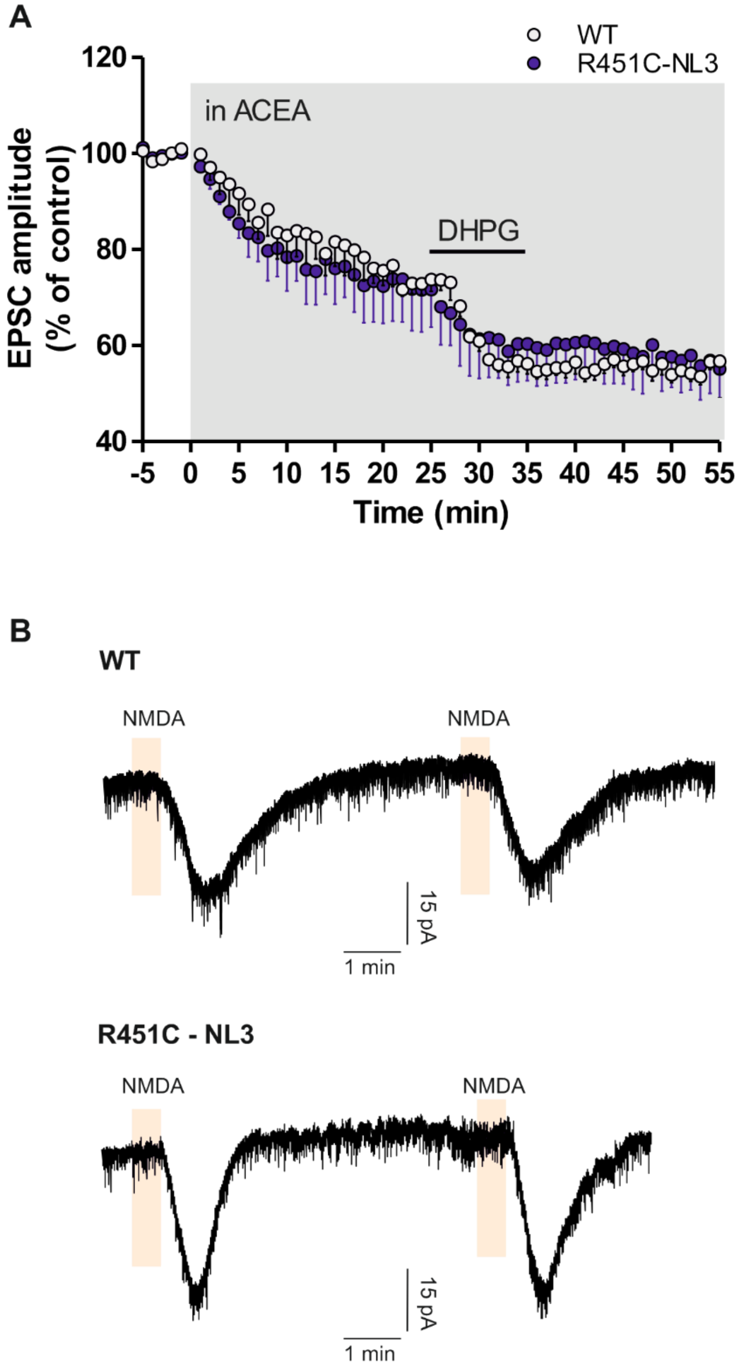

Supplementary Figure 2. (A). Time-course of EPSC amplitude recorded from WT and R451C-NL3 slices in the presence of 20 µM ACEA. After 25 minutes of incubation in ACEA, DHPG (50 µM, 10 min) was added to the perfusion solution. EPSC amplitude in ACEA (20-24'), WT: 73.62 ± 2.51 % of control, n = 4, R451C-NL3: 72.57 ± 8.57 %, n = 5; DHPG-LTD in ACEA (50-55'), WT: 55.22 ± 1.75 %, n = 4, R451C-NL3: 56.54 ± 6.72 %, n = 5; two-way ANOVA $F(1, 7)_{drug}$= 55.14, ****p =

0.0001; $F(1, 7)_{genotype} = 0.00$, $p = 0.9875$. (B). Representative traces of NMDA-mediated currents recorded from WT and R451C-NL3 slices, showing the absence of desensitization to 6-min apart brief bath applications of NMDA (30 µM, 30 s).

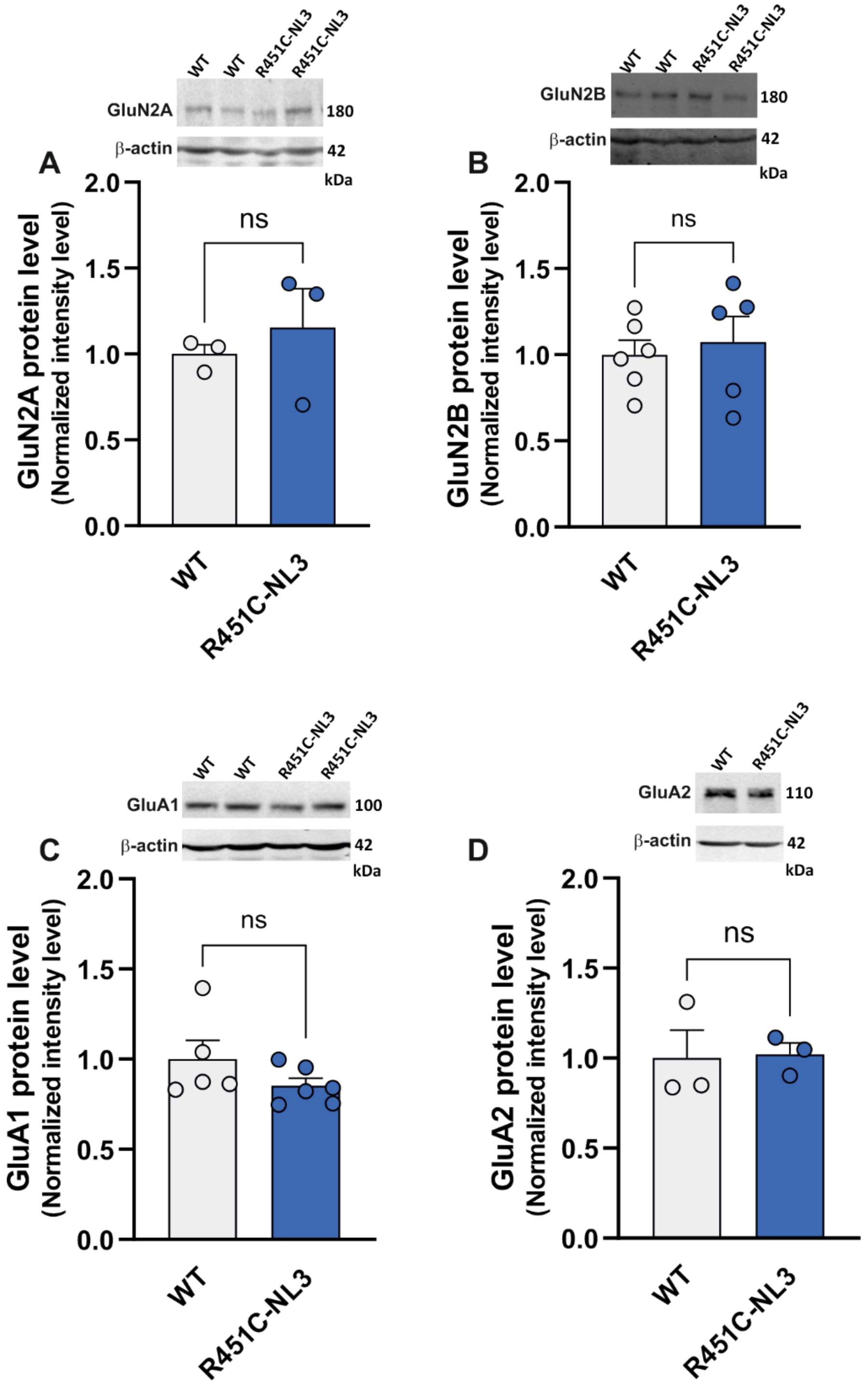

Supplementary Figure 3. Representative Western blots (*top*) and histograms (*bottom*) of (A) GluN2A, (B) GluN2B, (C) GluA1, (D) GluA2 receptor subunits protein levels in striatal lysates from R451C-NL3 and WT mice. Histograms report single measurements (dots), and mean ± SEM of data. (A) GluN2A, WT= 1.000 ± 0.053, N= 3; R451C-NL3= 1.155 ± 0.225, N= 3; unpaired t-test, t(4) =

0.667, $p = 0.541$; (B) GluN2B, WT= 1.000 ± 0.084, N= 6; R451C-NL3= 1.072 ± 0.152, N= 5; unpaired t-test, $t(9) = 0.436$, $p = 0.674$; (C) GluA1, WT= 1.000 ± 0.105, N= 5; R451C-NL3= 0.853 ± 0.042, N= 6; unpaired t-test, $t(9) = 1.399$, $p = 0.195$; (D) WT= 1.000 ± 0.156, N= 3; R451C-NL3= 1.021 ± 0.062, N= 3; Mann-Whitney test, $U = 3$, $p = 0.700$.

Impairment of group I metabotropic glutamate receptors in the dorsal striatum of the R451C-neuroligin 3 mouse model of Autism Spectrum Disorder

Maria Meringolo[1,2], Martina Montanari[1], Simona D'Antoni[3], Giuseppina Martella[1], Ilham El Atiallah[1], Giulia Ponterio[1], Annalisa Tassone[1], Ingrid Reverte[4], Daniele Caprioli[4], Georgios Strimpakos[5], Luisa Pieroni[2,6], Maria Vincenza Catania[3], Paola Bonsi[1*]

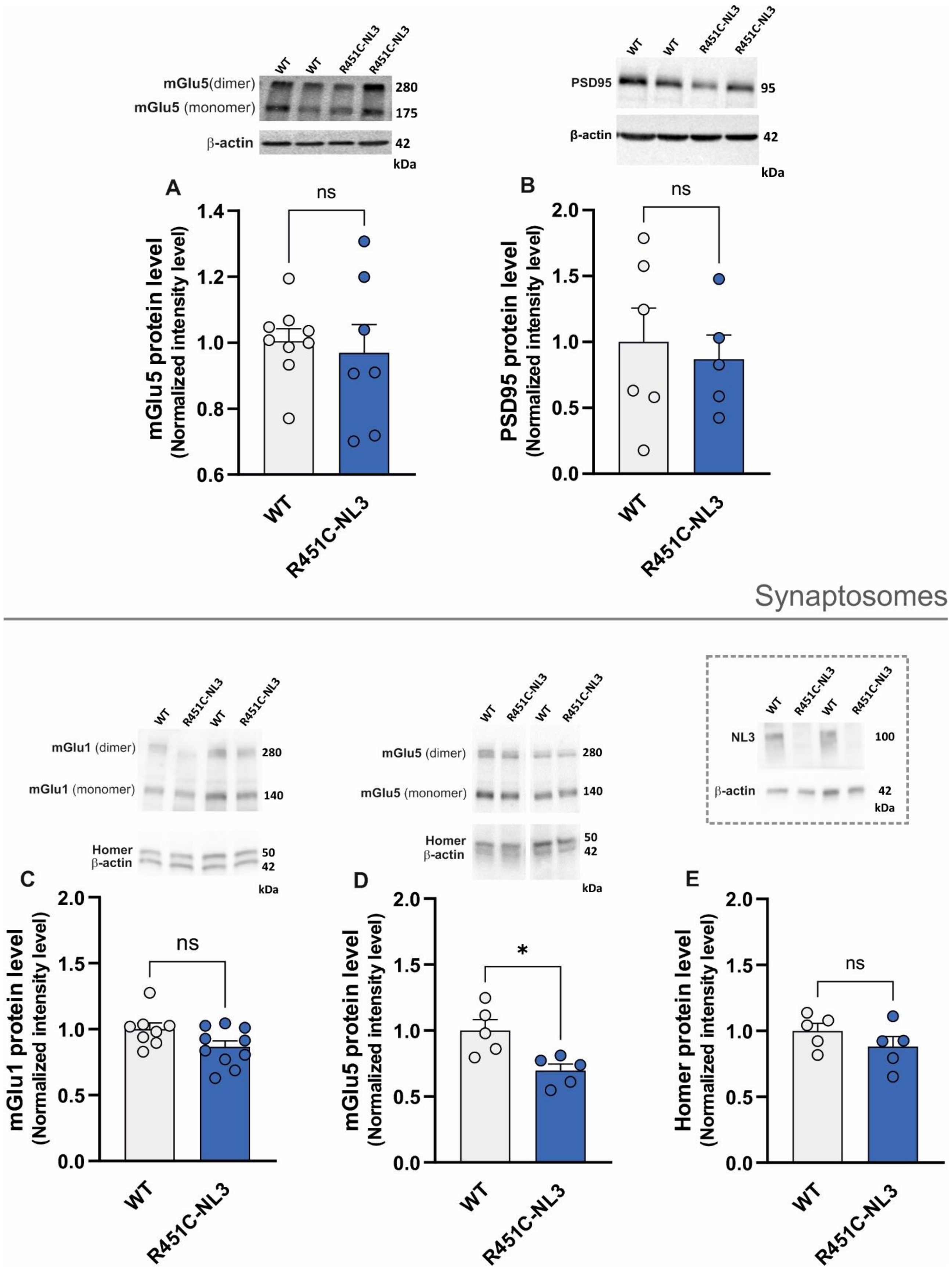

Maria Meringolo[1,2], Martina Montanari[1], Simona D'Antoni[3], Giuseppina Martella[1], Ilham El Atiallah[1], Giulia Ponterio[1], Annalisa Tassone[1], Ingrid Reverte[4], Daniele Caprioli[4], Georgios Strimpakos[5], Luisa Pieroni[2,6], Maria Vincenza Catania[3], Paola Bonsi[1*]

Supplementary Figure 4. (A,B) *Top*. Representative Western blot of (A) mGlu5 receptor and (B) PSD95 protein expression levels in cortical lysates of WT and R451C-NL3 mice. *Bottom*. Histograms reporting single measurements (dots), and mean ± SEM of data. (A) mGlu5, WT: 1.000 ± 0.037, n = 9, R451C-NL3: 0.968 ± 0.086, n = 7, unpaired t-test, t(14) = 0.410, p = 0.688. (B) PSD95, WT: 1.000 ± 0.258, n = 6, R451C-NL3: 0.869 ± 0.184, n = 5, unpaired t-test, t(9) = 0.396, p = 0.702. (C,D,E) *Top*. Representative Western blots of mGlu1 (C) or mGlu5 (D) receptor and Homer protein expression levels in cortical synaptosomal preparations from WT and R451C-NL3 mice, quantified in the *bottom* graphs. The *inset* shows a representative Western blot of NL3 quantification from cortical synaptosomal preparations. *Bottom*. Histograms report single measurements (dots), and mean ± SEM of data. (C) mGlu1, WT: 1.000 ± 0.047, n = 8, R451C-NL3: 0.865 ± 0.045, n = 10, unpaired t-test, t(16) = 2.048, p = 0.057. (D) mGlu5, WT= 1.000 ± 0.083, N= 5; R451C-NL3= 0.696 ± 0.050, N= 5; unpaired t-test, t(8) = 3.142, *p = 0.014. (E) Homer, WT= 1.000 ± 0.058 N= 5; R451C-NL3= 0.881± 0.076, N= 5; unpaired t-test, t(8) = 1.246, p = 0.248.

Impairment of group I metabotropic glutamate receptors in the dorsal striatum of the R451C-neuroligin 3 mouse model of Autism Spectrum Disorder

Maria Meringolo[1,2], Martina Montanari[1], Simona D'Antoni[3], Giuseppina Martella[1], Ilham El Atiallah[1], Giulia Ponterio[1], Annalisa Tassone[1], Ingrid Reverte[4], Daniele Caprioli[4], Georgios Strimpakos[5], Luisa Pieroni[2,6], Maria Vincenza Catania[3], Paola Bonsi[1*]

Figure 5E

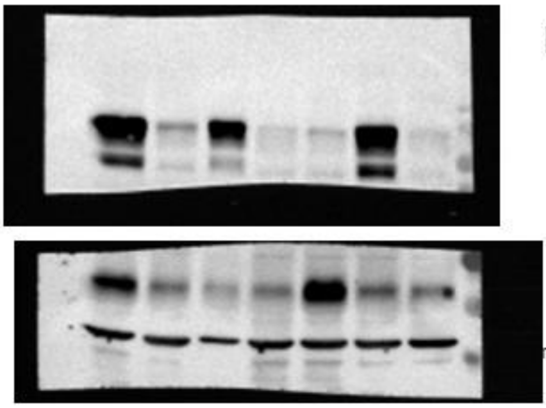

Figure 5A

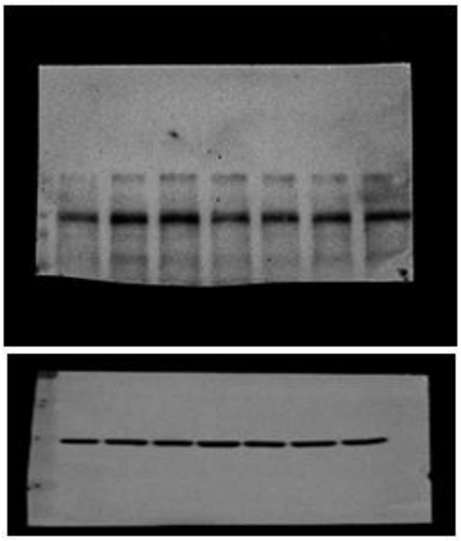

Figure 5B

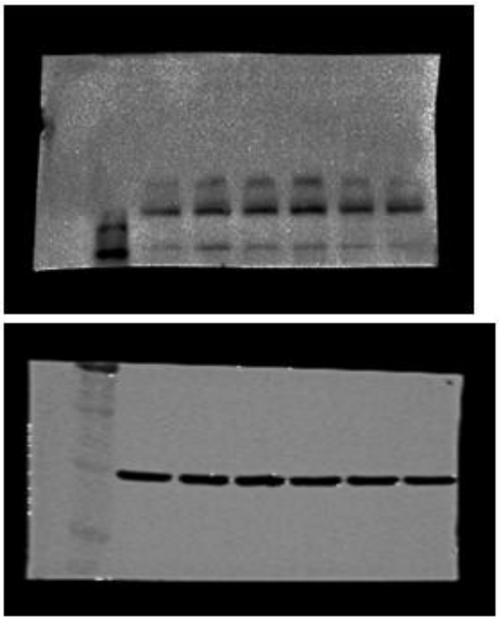

Figure 5C

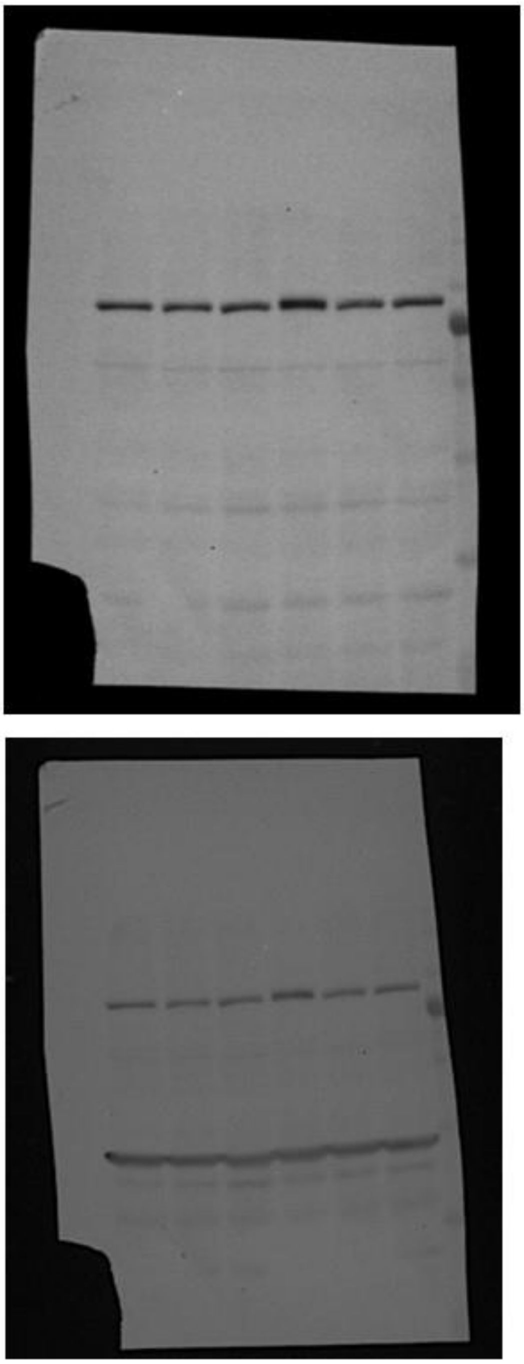

Impairment of group I metabotropic glutamate receptors in the dorsal striatum of the R451C-neuroligin 3 mouse model of Autism Spectrum Disorder

Maria Meringolo[1,2], Martina Montanari[1], Simona D'Antoni[3], Giuseppina Martella[1], Ilham El Atiallah[1], Giulia Ponterio[1], Annalisa Tassone[1], Ingrid Reverte[4], Daniele Caprioli[4], Georgios Strimpakos[5], Luisa Pieroni[2,6], Maria Vincenza Catania[3], Paola Bonsi[1*]

Figure 5D

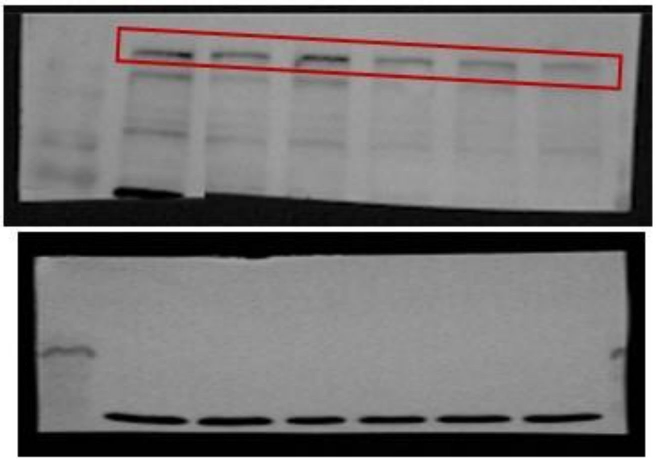

Suppl. Figure 3A

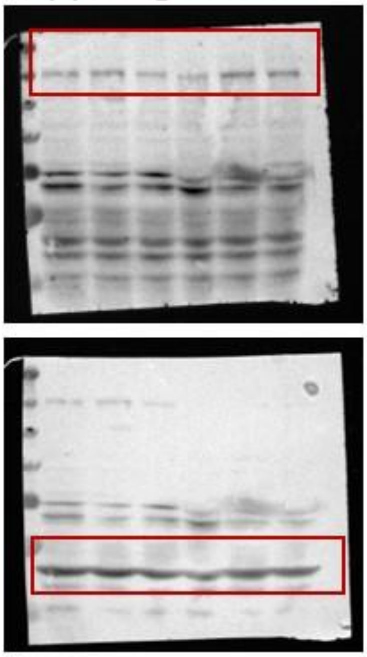

Suppl. Figure 3B

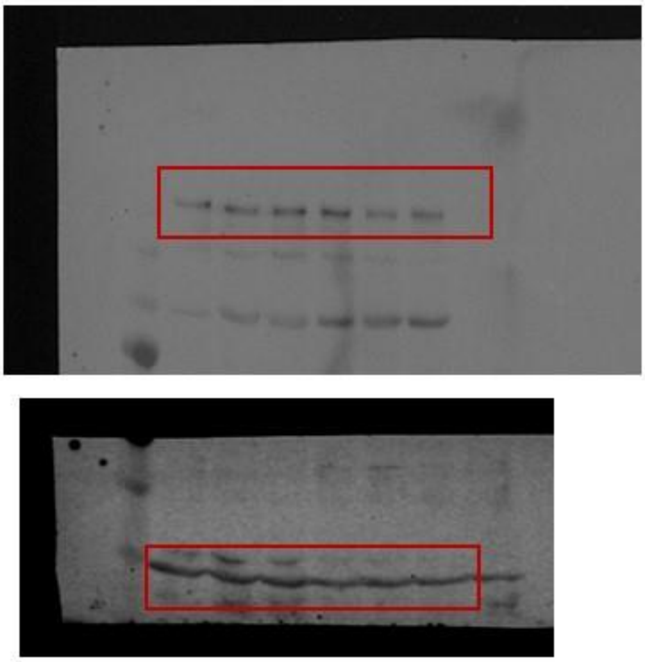

Suppl. Figure 3C

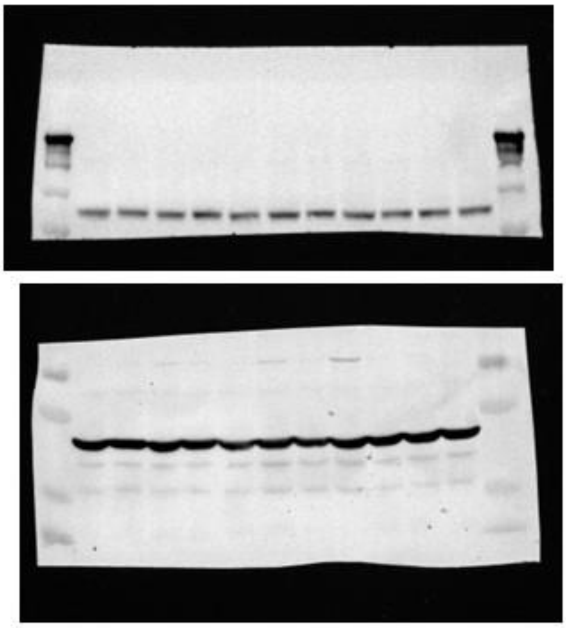

Suppl. Figure 3D

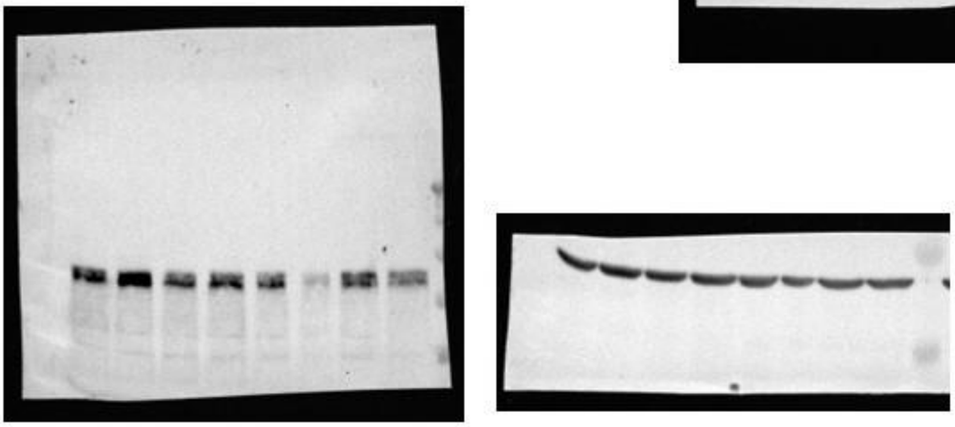

Impairment of group I metabotropic glutamate receptors in the dorsal striatum of the R451C-neuroligin 3 mouse model of Autism Spectrum Disorder

Maria Meringolo[1,2], Martina Montanari[1], Simona D'Antoni[3], Giuseppina Martella[1], Ilham El Atiallah[1], Giulia Ponterio[1], Annalisa Tassone[1], Ingrid Reverte[4], Daniele Caprioli[4], Georgios Strimpakos[5], Luisa Pieroni[2,6], Maria Vincenza Catania[3], Paola Bonsi[1*]

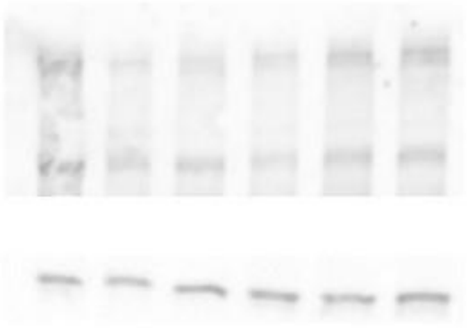

Figure 6A

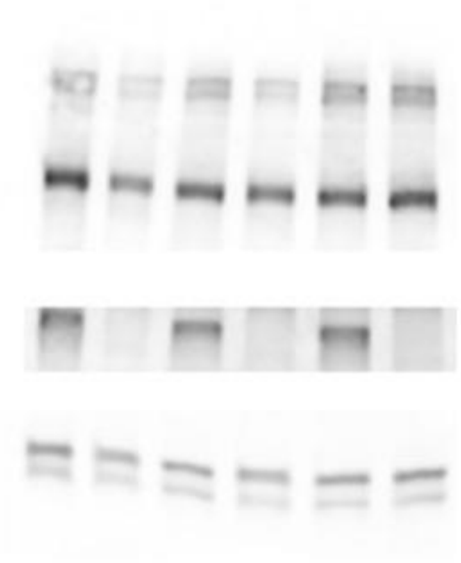

Figure 6B

Impairment of group I metabotropic glutamate receptors in the dorsal striatum of the R451C-neuroligin 3 mouse model of Autism Spectrum Disorder

Maria Meringolo[1,2], Martina Montanari[1], Simona D'Antoni[3], Giuseppina Martella[1], Ilham El Atiallah[1], Giulia Ponterio[1], Annalisa Tassone[1], Ingrid Reverte[4], Daniele Caprioli[4], Georgios Strimpakos[5], Luisa Pieroni[2,6], Maria Vincenza Catania[3], Paola Bonsi[1*]

Suppl. Figure 4A

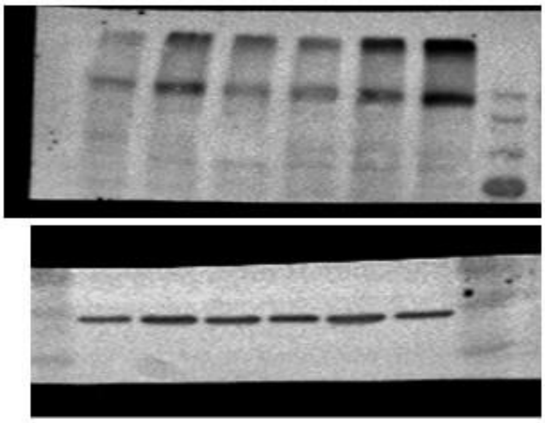

Suppl. Figure 4B

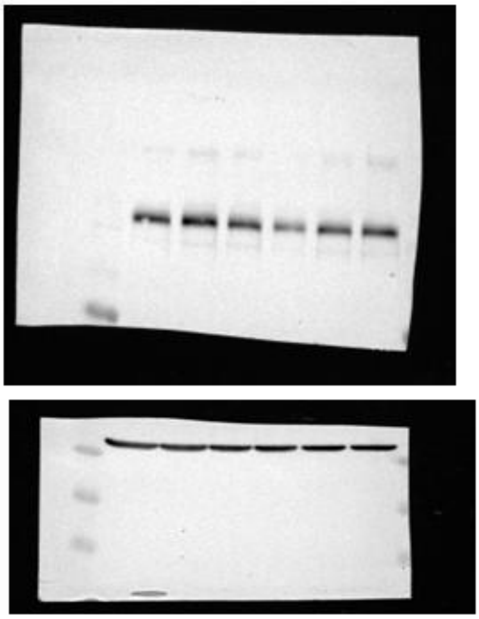

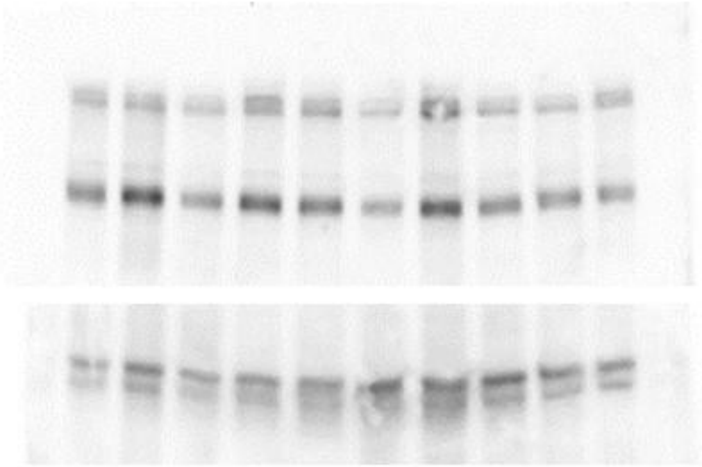

Suppl. Fig.4D

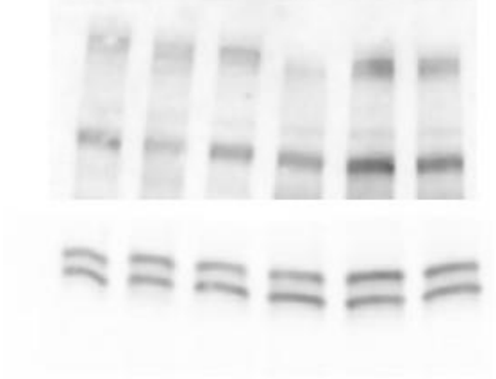

Suppl. Figure 4C

Insert Suppl. Figure 4

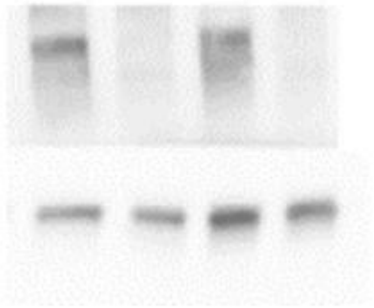

Supplement: Supplementary file 1 — Appendix S1: jnc70253‐sup‐0001‐AppendixS1.pdf. [file JNC-169-0-s001.pdf]
